# Alzheimer's disease‐associated R47H TREM2 increases, but wild‐type TREM2 decreases, microglial phagocytosis of synaptosomes and neuronal loss

**DOI:** 10.1002/glia.24318

**Published:** 2022-12-08

**Authors:** Alma S. Popescu, Claire A. Butler, David H. Allendorf, Thomas M. Piers, Anna Mallach, Julian Roewe, Peter Reinhardt, Alessandro Cinti, Loredana Redaelli, Christophe Boudesco, Laurent Pradier, Jennifer M. Pocock, Peter Thornton, Guy C. Brown

**Affiliations:** ^1^ Department of Biochemistry University of Cambridge Cambridge UK; ^2^ Neuroscience, BioPharmaceuticals R&D AstraZeneca Cambridge UK; ^3^ Department of Neuroinflammation UCL Queen Square Institute of Neurology London UK; ^4^ Neuroscience Discovery AbbVie Deutschland GmbH & Co. KG Ludwigshafen Germany; ^5^ Cell Biology Axxam SpA Milan Italy; ^6^ Rare and Neuro Diseases Sanofi R&D Chilly‐Mazarin France

**Keywords:** Alzheimer's disease, cystatin F, iPSC‐derived microglia, phosphatidylserine, R47H variant, synapses, TREM2

## Abstract

Triggering receptor on myeloid cells 2 (TREM2) is an innate immune receptor, upregulated on the surface of microglia associated with amyloid plaques in Alzheimer's disease (AD). Individuals heterozygous for the R47H variant of TREM2 have greatly increased risk of developing AD. We examined the effects of wild‐type (WT), R47H and knock‐out (KO) of human TREM2 expression in three microglial cell systems. Addition of mouse BV‐2 microglia expressing R47H TREM2 to primary mouse neuronal cultures caused neuronal loss, not observed with WT TREM2. Neuronal loss was prevented by using annexin V to block exposed phosphatidylserine, an eat‐me signal and ligand of TREM2, suggesting loss was mediated by microglial phagocytosis of neurons exposing phosphatidylserine. Addition of human CHME‐3 microglia expressing R47H TREM2 to LUHMES neuronal‐like cells also caused loss compared to WT TREM2. Expression of R47H TREM2 in BV‐2 and CHME‐3 microglia increased their uptake of phosphatidylserine‐beads and synaptosomes versus WT TREM2. Human iPSC‐derived microglia with heterozygous R47H TREM2 had increased phagocytosis of synaptosomes vs common‐variant TREM2. Additionally, phosphatidylserine liposomes increased activation of human iPSC‐derived microglia expressing homozygous R47H TREM2 versus common‐variant TREM2. Finally, overexpression of TREM2 in CHME‐3 microglia caused increased expression of cystatin F, a cysteine protease inhibitor, and knock‐down of cystatin F increased CHME‐3 uptake of phosphatidylserine‐beads. Together, these data suggest that R47H TREM2 may increase AD risk by increasing phagocytosis of synapses and neurons via greater activation by phosphatidylserine and that WT TREM2 may decrease microglial phagocytosis of synapses and neurons via cystatin F.

## Main Points

- R47H TREM2 variant, linked to Alzheimer's disease, increases neuronal loss and microglial phagocytosis likely via increased activation by phosphatidylserine.
- TREM2 increases cystatin F expression which inhibits general microglial phagocytosis.

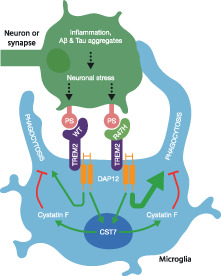

## 1 INTRODUCTION

Triggering receptor on myeloid cells 2 (TREM2) is a receptor expressed on myeloid cells that triggers an innate immune response when activated (Daws et al., 2003; Deczkowska et al., 2020). Ligands that activate TREM2 include amyloid beta (Aβ), negatively charged phospholipids such as phosphatidylserine, and lipoproteins such as APOE (Daws et al., 2003; Konishi & Kiyama, 2020; Vilalta et al., 2021). Activation of TREM2 modulates several microglial functions including inflammatory cytokine release, phagocytosis, proliferation, survival and differentiation (Deczkowska et al., 2020; Konishi & Kiyama, 2020). The main mechanism of TREM2 activation and signaling is via ligand binding to the extracellular domain, resulting in binding of TREM2 to DAP‐12, whose cytosolic ITAM (immunoreceptor tyrosine‐based activation motif) domains activate SYK tyrosine kinase, although TREM2 can also signal via binding DAP‐10 rather than DAP‐12 (Olufunmilayo & Holsinger, 2022).

Within the brain, TREM2 is mainly expressed on microglia, which are the primary immune cell of the central nervous system. TREM2 expression has been shown to be upregulated in microglia that associate with amyloid plaques in Alzheimer's disease (AD) (Brendel et al., 2017; Giraldo et al., 2013; Jay et al., 2015; Yuan et al., 2016). Genome wide association studies (GWAS) have identified several rare, heterozygous variants of TREM2 that increase risk of AD, including R47H TREM2, which increases AD risk about four‐fold (Giraldo et al., 2013; Guerreiro et al., 2012; Jonsson et al., 2013; Olufunmilayo & Holsinger, 2022). Understanding how this single mutation confers this considerably increased risk of AD when present on only one allele, may give important insights into the mechanism of AD and how to prevent it.

The R47H mutation is near the ligand binding site of TREM2 and may reduce binding of some ligands to TREM2 and reduce clustering of microglia around Aβ plaques (Kober et al., 2016; Sudom et al., 2018). This has led to the idea that R47H is a loss‐of‐function mutation, which in turn suggests that wild‐type TREM2 protects against AD, probably by increasing microglial phagocytosis of Aβ, and thereby removing neurotoxic Aβ oligomers and compacting amyloid plaques (Condello et al., 2015; Gratuze et al., 2018; Kulkarni et al., 2021; Olufunmilayo & Holsinger, 2022; Yuan et al., 2016). However, the finding that the R47H mutation reduces binding of some ligands (such as Aβ) does not preclude the possibility that it also increases the binding and/or activation of TREM2 by other ligands, such as phosphatidylserine. Establishing whether TREM2 R47H is linked to AD due to a loss‐of‐function or gain‐of‐function is important, because if R47H causes a loss‐of‐function, this would imply that WT TREM2 is beneficial in AD and thus stimulating WT TREM2 may be beneficial. If, however, the R47H variant increases TREM2 function, then inhibiting WT TREM2 could be beneficial. Whether it is gain or loss of function could also be context/ligand dependent.

Triggering receptor on myeloid cells 2 acts as a phagocytic receptor on microglia by binding to ligands, such as exposed phosphatidylserine on apoptotic cells or neuronal debris, and then activating engulfment of these targets via DAP‐12 and SYK (Charles et al., 2008; Deczkowska et al., 2020; Takahashi et al., 2005). However, phosphatidylserine can also be exposed on live synapses and neurons in some circumstances, resulting in phagocytosis of these live synapses or neurons via various phagocytic receptors, including TREM2 (Neher et al., 2011; Neniskyte et al., 2011; Scott‐Hewitt et al., 2020). TREM2 appears to also mediate microglial phagocytosis of synapses during development (Filipello et al., 2018), aging (Linnartz‐Gerlach et al., 2019; Qu & Li, 2020) and AD (Sheng et al., 2019).

Expression or activation of TREM2 can also reprogram microglia via inducing a Disease Associated Microglia (DAM) expression profile (Keren‐Shaul et al., 2017). This DAM expression profile is observed in microglia in multiple disease states, including AD and AD mouse models (Butovsky & Weiner, 2018; Keren‐Shaul et al., 2017), but the effects of this expression profile on microglial functions are unclear. The DAM profile includes reduced expression of homeostatic genes *P2ry12*, *Tmem119*, *and Cx3cr1*, and increased expression of genes regulating phagocytosis, including *Itgax, Axl, ApoE*, and *Cst7* (Keren‐Shaul et al., 2017). *Cst7* is the gene for the protein cystatin F, which is highly upregulated by TREM2 (Keren‐Shaul et al., 2017). Cystatin F is an inhibitor of cysteine proteases in the lysosomes, including cathepsins C, H & L, the inhibition of which can result in reduced lysosomal function and microglial phagocytosis (Kang et al., 2018; Ofengeim et al., 2017). Thus, there is a possibility that TREM2 could inhibit general phagocytosis via inducing cystatin F, while at the same time inducing phagocytosis of TREM2‐specific targets. TREM2 expression has been shown to inhibit the phagocytosis of a variety of targets, but the mechanism is unclear (Carrillo‐Jimenez et al., 2018; Schoch et al., 2021).

In this study, we used cultured BV‐2, CHME‐3 and iPSC‐derived microglia to show that expression of R47H TREM2 in microglia increases phagocytosis of synapses and increases neuronal loss, indicating a gain‐of‐function relative to wild‐type TREM2, which might contribute to the increased AD risk conferred by R47H TREM2.

## 2 MATERIALS AND METHODS

### 2.1 General cell lines and maintenance

BV‐2 and CHME‐3 cells were cultured in Dulbecco's Modified Eagle Medium (DMEM) (life technologies, 41965039), HEK and SY5Y cells were cultured in DMEM:F12 (Life technologies, 31331028), and all cell lines were supplemented with 10% heat inactivated FBS (hiFBS) (life Technologies, 10500064) and 1% penicillin/streptomycin (pen/strep) (Sigma, P4333). All cell lines were stored in an incubator set at 37°C and 5% CO_2_. The maximum passage of all cell lines used was 25.

### 2.2 Cloning and site directed mutagenesis of human TREM2 transfer lentivirus plasmids

Human TREM2 ORF (NM_018965.3) on a pcDNA3.1 vector with a C‐terminal FLAG‐tag was purchased from Genscript. The R47H variant of TREM2 was generated by site‐directed mutagenesis using the QuickChange© kit (Agilent). Both WT and R47H TREM2 variant were subcloned into modified pWPI (Allendorf et al., 2020) and transfected with lentiviral packaging and envelope vectors into HEK293T cells to generate lentiviral particles.

### 2.3 Lentiviral production and transduction

Packaging, psPAX2 (12260), envelope, pMD2.G (12259) and eGFP only (control) (12254) were all sourced from Addgene. All plasmids were sent to the Department of Biochemistry Sanger Sequencing lab to ensure all sequences were correct before any experiments were conducted. Packaging, envelope and TREM2 transfer plasmids were combined according to the Addgene lentiviral production protocol. The plasmid complexes were then transfected into HEK 293 T cells using polyethylenimine (PEI) (Sigma, 408727) transfection reagent in a 3:1 ratio of PEI:DNA. After 24 h, media was exchanged. After a further 24 h, media was harvested, centrifuged and filtered through a 0.45‐micron filter. These media (containing lentiviral particles) were then added to BV‐2 or CHME‐3 cells at 50%–60% confluency with the addition of 8 μg/ml polybrene (Merck, TR‐1003‐G). After 18 h, cells were harvested and eGFP expression was assessed using flow cytometry (Cytoflex, Beckman). Cells that were positive for eGFP were Fluorescence‐Activated Cell Sorting (FACS) (Aria III) sorted by the University of Cambridge Flow Cytometry unit located in the Department of Pathology, collecting only the highest 25% of eGFP‐expressing cells, and therefore the highest, eGFP only, eGFP‐WT‐TREM2 and eGFP‐R47H‐TREM2 expressing cells were used for experiments.

### 2.4 Isolation and maintenance of primary mouse cerebellar granule cells (CGCs) cultures

For mixed neuron‐glia cultures, which comprise of ~85% neurons, 10% microglia and 5% astrocytes. Neonatal mice aged between 3–5 days were decapitated, according to schedule 1 culling procedures and protocol found in Carrillo‐Jimenez et al. (2018) was followed. Briefly, the whole brain was extracted and the cerebellum was dissected. Meninges were removed under a microscope and the cerebellum was then cut into small pieces using a scalpel and placed in 1X versene (Thermofisher, 15040066), which is a milder dissociation reagent compared to trypsin. Tissue was incubated for 5 min at 37°C. Using a cut blue (1000 μl) pipette tip the tissue was dissociated using three up and down pipette motions. The non‐ disaggregated tissue was allowed to settle and the versene containing dissociated cells (which was slightly cloudy) was removed and placed into 25 ml of CGC media (DMEM supplemented with 5% horse serum, (Invitrogen, 26050088), 5% performance plus FBS, 5 mM 4‐(2‐hydroxyethyl)‐1‐piperazineethanesulfonic acid (HEPES) (Melford laboratories, H75030), 20 mM KCl (sigma, 31,246), 2 mM L‐glutamine (sigma, G8530), 13 mM glucose (Sigma, 16325) and 1% pen/strep). This process was repeated three times and repeated a further three times using an uncut blue tip. Once all tissue had been dissociated and placed into CGC media the cells were centrifuged for 7 min at 950 rpm with slow start‐stop. The supernatant was then removed, and the pellet resuspended in CGC media. The cell suspension was then filtered through a 40‐micron strainer, live cells counted using trypan blue (sigma, T8154) and a hemocytometer and then cells seeded in pre‐coated 48 well plates at a density of 1 × 10^6^ cells per ml (250 μl was added to each well). 48‐well plates were pre‐coated with 0.001% poly‐L‐lysine for at least 30 min at room temperature and then washed thoroughly with ddH_2_O and PBS. 24 h after seeding, cells were given a full media swap with CGC media, to remove dead cells and debris. The neuronal‐glial cultures were used for experiments after 7 days in vitro.

### 2.5 Co‐culture of BV‐2 cells and primary mouse neuronal‐glial cultures

On day 7 in vitro, the neuronal‐glial cultures (250,000 cells/well/250 μl culture media) were co‐cultured with 50,000 BV‐2 cells, or media only, for 24 h. Briefly, BV‐2 cells were cultured in T‐75 flasks in high serum media (10% FBS, 1% pen/strep, DMEM) until they reached ~80% confluency, media was then aspirated, cells washed in PBS and detached using 1X trypsin for 3 min at 37°C. Trypsin was quenched using high serum media and cells centrifuged for 5 min at 150 g. Pellet of cells was resuspended in 2 ml high serum media and counted with trypan blue and hemocytometer. BV‐2 cells were then diluted in CGC media to 1 × 10^6^ cells per ml and 50 μl of BV‐2 cell suspension, or media only, +/−100 nM annexin V (BioVision) was added per well to neuronal‐glial cultures. After 24 h, cells were labeled with the nuclear stain Hoechst 33342 (10 μg/ml), a marker of necrosis propidium iodide (1 μg/ml) and the microglia‐specific marker Alexa Fluor 488‐tagged isolectin B4 (IB4, 2 μg/ml) for 20 min. Cells were then imaged on an EVOS fluorescence microscope (Thermofisher) and the number of microglia, apoptotic, necrotic and healthy neurons were counted on the basis of their staining and morphology using FIJI‐ImageJ.

### 2.6 Human iPSC‐derived microglia (iPS‐Mg) derivation and culturing

Ethical permission for this study was obtained from the National Hospital for Neurology and Neurosurgery and the UCL Queen Square Institute of Neurology joint research ethics committee (study reference 09/H0716/64). R47Hhet fibroblasts from two different patients were obtained from Prof M. Blurton‐Jones through a material transfer agreement with the University of California Irvine, Alzheimer's Disease Research Center. The following iPSC lines were used: two TREM2 R47Hhet patient lines (ADRC8.6 and 26.15), the R47Hhom BIONio10‐C7 line (EBiSC) and one TREM2 common/WT variant line, BIONi010‐C (EBiSC).

Human iPS‐Mg were generated using our previously published protocol and iPSC lines (Piers et al., 2020; Xiang et al., 2018). Briefly, embryoid bodies were generated from iPSC by addition of IL‐3, MCSF and β‐mercaptoethanol using a previously described protocol (Garcia‐Reitboeck et al., 2018; van Wilgenburg et al., 2013). Myeloid progenitors were further differentiated using IL‐34, MCSF and TGF‐β for 2 weeks, in addition to CX3CL1 and CD200 for the final 3 days (Piers et al., 2020; Xiang et al., 2018). The iPS‐Mg thus generated were previously characterized in our laboratory with regard to their expression of the so called “microglial signature genes” (Butovsky & Weiner, 2018), and also display typical microglial functions, such as phagocytosis of particles, intracellular signaling, and responses to inflammatory stimuli (Cosker et al., 2021; Piers et al., 2020).

### 2.7 Measuring TREM2 activation of pSYK in human iPSC‐derived microglia

Liposomes were prepared as described in (Boudesco et al., 2022) in a 50/32/18 molar ratio of cholesterol/ 1,2‐dioleoyl‐sn‐glycero‐3‐phosphocholine/phospholipid respectively, where the phospholipid was either 1,2‐dioleoyl‐sn‐glycero‐3‐phospho‐L‐serine (phosphatidylserine liposomes) or 1,2‐dioleoyl‐sn‐glycero‐3‐ phosphocholine (phosphatidylcholine liposomes). Chloroform‐dissolved lipids (purchased from Avanti) were mixed in 0.5 ml of absolute ethanol, and solvents removed by rotary evaporation at 45°C under gradually lowered pressure exposure (from 999 mbar to 0). The remaining lipid film was dried under vacuum at room temperature overnight, then hydrated in PBS for 30 min at 45°C with agitation to produce a multilamellar lipid solution of 1.75 mg/ml. The flask was sonicated in an ultrasonic water bath for 2 min at 25°C, and then an immersed ultrasonic probe tip (Vibracell 75115, Bioblock Scientific, 500 W), using a 10 s pulse with a 30 s interval between 6 pulses, at 25°C and 25% power. The resulting emulsion was then extruded by passaging 2 ml samples 11 times through a mini extruder (Avanti Polar Lipids) equipped with a polycarbonate membrane (pore size 100 nm). Liposome solution was stored at 4°C until further use.

Human iPSC‐derived microglia were generated according to the protocol described by Haenseler et al. (2017) using the following iPSC lines: BIONi010‐C (isogenic control line) and BIONi010‐C‐17 (TREM2 KO) both obtained from EBiSC (https://cells.ebisc.org/search). Only precursors from harvest week 6 to harvest week 13 were collected to undergo differentiation into microglia by culturing them for 14 days in Advanced DMEM/F12 (ThermoFisher, #12634‐010) supplemented with 1 mM Glutamax (ThermoFisher, #35050‐038), 100 U/ml penicillin and 100 μg/ml streptomycin (ThermoFisher, #15140‐122), 50 μM 2‐mercaptoethanol (ThermoFisher, #31350‐010), 100 ng/ml IL‐34 (PeproTech, #200‐34) and 10 ng/ml GM‐CSF (ThermoFisher, #PHC2015) in uncoated 384‐well plates (Greiner #781091) at a density of 7500 cells/well in a volume of 40 μl medium/well. Half of medium was discarded and replaced twice per week. On day 14 of microglia differentiation, cells were tested for TREM2 activation. The remaining medium present on the cells was supplemented with a 2x liposome solution of respectively DOPS or DPPC in microglia medium. The microglia were incubated with the liposomes for 2 min at room temperature. As a control, microglia were treated with the TREM2 agonistic antibody (R&D systems, #AF1828) for 5 min at room temperature.

Phosphoprotein detection in cell lysates after stimulation was performed by AlphaLISA technology according to manufacturer's instructions (AlphaLISA® SureFire® Ultra™, Perkin Elmer), using P‐Syk 525/526 (ALSU‐PSYK‐10K) and SYK (ALSU‐SYK‐10K), kits. Briefly, the microglia were incubated with the liposomes for 2 min at room temperature. As a control, microglia were treated with the TREM2 agonistic antibody (R&D systems, #AF1828) for 5 min at room temperature. After incubation, all the medium was removed from the iPSC‐derived microglia, and cells were lysed with the AlphaLISA lysis buffer solution (prepared according to the manufacturer's instructions) for 10 min on a shaker (350–400 rpm) at room temperature. AlphaLISA assays were performed in white 384‐well AlphaPlates (PerkinElmer #6005350) according to the manufacturer's protocol. In brief, 10 μl of fresh cell lysate was transferred to a 384‐well Alphaplate followed by the addition of 5 μl of Acceptor mix. After 1 h of incubation at room temperature 5 μl Donor mix was added in a light reduced environment, followed by incubation for 1 h protected from light. Plate reading was done using the PHERAstar FSX plate reader (BMG LABTECH). All samples were run in technical quadruplicates. For data analysis, the average of the technical quadruplicates was calculated per sample. pSYK levels were normalized to the relative Total SYK levels.

### 2.8 Differentiation of LUHMES neuronal‐like cell line

T‐175 cm^2^ tissue culture treated flasks and 24‐well tissue culture plates were coated with poly‐L‐ornithine (sigma, P3655) overnight at 37°C. After this, plates and flasks were washed thoroughly with ddH_2_O and then coated with 1 μg/ml fibronectin for minimum 3 h at 37°C. After 3 h, plates and flasks were washed thoroughly in ddH_2_O and left to dry in a tissue culture hood. Coated plates and flasks were either used immediately once dry or kept sterile at 4°C and used within 14 days of coating. LUHMES were maintained in DMEM: F12 media (Lonza, BE‐12‐7191), supplemented with 100X N2 reagent (Thermofisher, 17502048), 1% pen/strep and 40 ng/ml human basic fibroblast growth factor (bFGF) (Thermofisher, 13256‐029). For differentiation, cells were dissociated from maintenance flasks using 1X TrypLE CTS select enzyme dissociation reagent (Thermofisher, A1285901), quenched in growth media and centrifuged at 1000 rpm, for 5 min. Cells were stained with trypan blue to identify the extent of cell death and counted using a hemocytometer. For experiments, LUHMES were seeded at 200,000 per ml in differentiation media (500 μl of differentiation media was added per well). Differentiation media comprised of DMEM: F12 media, supplemented with 100X N2 reagent, 1% pen/strep, 2 ng/ml glial cell line‐derived neurotrophic factor (GDNF) (Miltenyi biotec, 130‐096‐290), 250 μM dibutyryl‐cyclic adenosine monophosphate (db‐cAMP) (Selleckchem, S7858) and 1 μg/ml tetracycline (sigma, T7660). After 1 day in differentiation media the media was fully swapped, to remove potential debris which may have been generated from the seeding process. A further full media swap was conducted on Day 4. On day 5, LUHMES were deemed fully differentiated after comparing mRNA levels of key neuronal markers, neuronal processes seen via imaging and from previous literature experience.

### 2.9 Co‐culture of LUHMES with CHME‐3 cells

On day 4 of differentiation, LUHMES were stained with 5 μM live CellTrace Violet (Thermofisher, C34557) for 10 min at 37°C. Cells were washed three times in DMEM: F12 media and then fresh differentiation media added. On day 5, after full differentiation had occurred, TREM2 overexpressing CHME‐3 cells were added to LUHMES at a 1:1 ratio (i.e., 100,000 CHME‐3:100,000 LUHMES) in DMEM supplemented with 0.5% hiFBS. After 3–4 h, co‐cultures were treated with media only or 2 μM synthetic monomeric Aβ 1–42 (Anaspec, AS‐20276). After 48 h, cultures were imaged on EVOS fluorescence microscope (Thermofisher) and number of blue stained nuclei‐like cells were counted using automated counting software, Qupath.

### 2.10 Proliferation assay

1 × 10^6^ CHME‐3 or 500,000 BV‐2 cells were seeded in a T‐75 cm^2^ flask and cultured in 10 ml DMEM supplemented with 10% hiFBS and 1% pen/strep for 48 h or 24 h, respectively. Cells were then harvested with trypsin, stained with trypan blue (to assess cell death) and counted using a hemocytometer.

### 2.11 Synaptosome preparation and labeling

Rat cortical synaptosomes were isolated as in Dunkley et al., 2008. Synaptosomes were labeled with 10 μM pHrodo succinimidyl ester (ThermoFisher), and then washed to remove unbound pH‐Rodo. PHrodo fluoresces when the labeled synaptosomes are phagocytosed into the acid environment of lysosomes.

### 2.12 Phagocytosis assays

A total of 10,000 CHME‐3 cells, or 25,000 BV‐2 microglia, were seeded in 0.5% FBS DMEM in 96‐well plates, in triplicate 24 h before adding targets. Cells were pre‐treated +/−10 μM cytochalasin D (CytoD) for 1 h as a negative control for phagocytic uptake. Cells were then incubated at 37°C with a phagocytic target; 16 μg pHrodo‐labeled synaptosomes for 1 h (CHME‐3) or 1.5 h (BV‐2), 40,000 5 μm Sky‐Blue carboxyl fluorescent beads (Spherotech, CFP‐5070‐2) for 2 h or 3 μm Rhodamine phosphatidylserine lipid beads (Echelon Biosciences, P‐B1PSRh, custom made) for 1 h (CHME‐3) or 1.5 h (BV‐2). All cells were then harvested using trypsin and resuspended in 1X PBS and analyzed on a flow‐cytometer; CytoFLEX (Beckman) for CHME‐3 samples and Attune NxT (Thermofisher) for BV‐2 samples.

For iPS‐Mg, 25–50,000 cells were seeded onto 24 well plates. iPS‐Mg were incubated with 16 μg rat cortical synaptosomes for 1–2 h at 37°C in a tissue culture incubator. Cells were washed and gently scraped into 80 μl of PBS (w/o Ca^2+^/Mg^+^). Three wells were combined and analyzed by flow cytometry, using a BD FACs Calibur machine and post‐analysis with Flowing software.

### 2.13 Flow cytometry

At least 5000 events were analyzed for each treatment replicate. FlowJo (version 10) was used for creating raw histograms and flow plots. Forward and side scatter was used to distinguish cells from unphagocytosed targets, that is, beads and synaptosomes, by gating on cells in the forward and side scatter plots. Within this scatter gate, a fluorescence gate was set to identify cells that were over a threshold of fluorescence. For phagocytic targets, this fluorescence gate was set so that for cells incubated in the absence of any fluorescent targets (i.e., absence of fluorescent beads and synaptosomes), 99% of the cells were below this gated fluorescence and 1% were above this gate. So, for cells incubated in the presence of fluorescent targets, the % of cells with fluorescence greater than the gated fluorescence were used as the measure of the % of cells that had phagocytosed the targets. Mean Fluorescence Intensity (MFI) was also used for certain phagocytosis assays. Data shown as MFI has had untreated and CytoD controls subtracted to highlight active phagocytosis MFI. For CHME‐3 samples the CytoFLEX (Beckman) flow‐cytometer was used: eGFP was detected using the 488 nm excitation laser and 525/40 filter and PE and pH‐rodo was detected using the 561 nm laser and 585/42 filter. For BV‐2 samples the Attune NxT (Thermofisher) flow‐cytometer was used: eGFP was detected using the 488 nm laser and 530/30 filter, PE and pH‐rodo was detected using the 561 nm laser and 585/16 filter, PE/Dazzle™594 annexin V (BioLegend, 640955) was detected using the 561 nm laser and 620/15 filter and sky blue fluorescent beads were detected using the 405 nm laser and VL1 filter. For quantification of TREM2 protein on the surface of cells, forward and side scatter was used to identify cells, and the PE fluorescence of IgG control‐PE treated cells was used to gate the fluorescence of anti‐TREM2‐PE‐treated cells.

### 2.14 Lipofectamine 3000 mediated RNAi knock down of human CST7 in CHME‐3 cells

A total of 3 × 10^5^ cells were seeded (in 1 ml of media) in a 6‐well plate in DMEM supplemented with 10% hiFBS and 1% pen/strep. After 24 h cells were subjected to RNAi:lipid mix containing 60 pmol human CST7 (Thermofisher, 4392420) targeting or non‐target (NT) (scrambled RNAi) (Thermofisher, 4390843) and 3% (v/v) lipofectamine 3000 (Thermofisher, L3000001) in serum free OptiMEM media (Table 1). After 3 h incubation at 37°C, transfection media was removed and replaced with DMEM supplemented with 10% hiFBS and 1% pen/strep. After a further 24 h, cells were harvested using trypsin and seeded into phagocytosis plates either 96‐well or 24‐well plates, assay depending, in DMEM supplemented with 0.5% hiFBS and 1% pen/strep. RNA was extracted 48 h post transfection for analysis via RT‐qPCR. Phagocytosis assays were performed 48 h post transfection.

**TABLE 1:** Summary of RNAi

Target gene	Sequence	Supplier	Catalog no./assay ID
Non‐target (scrambled)	Unknown (protected sequence)	Thermofisher	4390843
Human CST7	Sense: GAUACAGUGUUGAAAAGUUTT Anti‐sense: AACUUUUCAACACUGUAUCTG	Thermofisher	4392420/s16218

*Note*: Target gene name, sequences and catalog numbers of the RNAi that were used in this study.

### 2.15 RNA extraction and reverse transcription

Whole RNA was extracted using the Monarch total RNA Miniprep kit and associated protocol (NEB, T2010). Briefly, cells were lysed and run through spin columns to remove genomic DNA and to purify RNA. RNA was eluted from columns using nuclease free H_2_O (Severn Biotech Ltd, 20‐9104‐05). Concentration of RNA was quantified on nanodrop (Thermofisher). cDNA was synthesized using 1 μg whole RNA, 1 μl of 10 mM deoxyribose nucleotide triphosphates (DNTPs) (Thermofisher, R0192) and 1 μl of 20X random hexamer primers (ThermoFisher, SO142). Samples were made up to 13 μl with nuclease free water. RNA, DNTPs and random hexamer primers were incubated at 65°C for 5 min, before being placed on ice. After ~1 min, 2 μl of 100 mM 1,4‐dithiothreitol (DTT) and 4 μl 5X first strand buffer (250 mM tris(hydroxymethyl)aminomethane (Tris)‐HCl (pH 8.3), 375 mM KCl, 15 mM MgCl_2_) were added per sample. Samples were left at room temperature for ~3 min before adding 1 μl (200 units) of Superscript II Transcriptase (Invitrogen, 18064022) was then added per sample. Total volume of sample was 20 μl and these were placed in a thermocycler (Biorad) and cDNA generated using the following conditions: 25°C for 10 min, 42°C for 50 min, 72°C for 15 min, 4°C hold.

### 2.16 Quantitative PCR (qPCR)

Quantitative PCR was performed using SYBR Green JumpStart Taq ReadyMix (Sigma, S4438). Briefly, a master mix for each gene containing: 12.5 μl of SYBR Green, 2 μl of 10 μM primers (Table 2) and 9.5 μl of nuclease free water (per sample) was made. Of that master mix, 90 μl was aliquoted out for each cDNA sample. 3.75 μg of cDNA was added to the 90 μl master mix and vortexed well. From this, samples were loaded into Qiagen 0.1 ml PCR strip tubes, in triplicate, for amplification. Amplification was performed using Rotor‐gene Q cycler (Qiagen) with the following parameters: 95°C for 10 min, 40 cycles of 95°C for 10 s, 60°C for 15 s, 72°C for 20 s, followed by 65°C melt. All samples were normalized to one or more housekeeping genes and then represented as a fold‐change relative to the respective control cell‐line.

**TABLE 2:** Summary of qPCR primers

Gene	Species	Sequences
GAPDH	Mouse	Forward: GTTGTCTCCTGCGACTTCA Reverse: GGTGGTCCAGGGTTTCTTA
GusB	Mouse	Forward: GTTGAGGATCAACAGTGCCC Reverse: ATGTCAGCCTCAAAGGGGAG
18s rRNA	Human	Forward: GGCCCTGTAATTGGAATGAGTC Reverse: CCAAGATCCAACTACGAGCTT
TREM2	Mouse	Forward: GACCTCTCCACCAGTTTCTC Reverse: TACATGACACCCTCAAGGA
TREM2	Human	Forward: ATGATGCGGGTCTCTACCAGTG Reverse: GCATCCTCGAAGCTCTCAGACT
DAP12	Mouse	Forward: GTTGACTCTGCTGATTTGCCCT Reverse: CCCTTCCGCTGTCCCTTGA
DAP12	Human	Forward: TGGTGCTGACAGTGCTCATTGC Reverse: CTGATAAGGCGACTCGGTCTCA

*Note*: Target gene name, species and sequences of all qPCR primers used throughout this study. All were purchased from sigma.

### 2.17 Protein surface expression staining

For detection of human TREM2 on CHME‐3 cells and BV‐2 cells, rat anti‐human/mouse TREM2 (clone: 237920) conjugated to Phycoerythrin (PE) fluorophore (R&D systems, FAB17291P) and isotype control, monoclonal rat IgG2B (clone: 141945) conjugated to PE fluorophore (R&D systems, IC013P) were used. Briefly, antibodies were diluted 1:50 (working concentration of 0.5 μg/ml) in ice‐cold flow cytometry staining buffer 1X (R&D Systems, FC001), added to tubes containing 100,000 cells/tube and kept in the dark at 4°C (to prevent receptor internalization) for 30 min. Cells were then washed three times with 1X PBS, resuspended in 1X FACS staining buffer and PE fluorescence quantified using a flow cytometer CytoFLEX (Beckman) for CHME‐3 samples and Attune NxT (Thermofisher) for BV‐2 samples. For BV‐2 microglia, an additional step was required to reduce non‐specific binding. Mouse Seroblock FcR (Biorad, FCR4G8) diluted 1:50 in FACS staining buffer was added to mouse BV‐2 microglia for 20 min on ice prior to adding antibodies.

### 2.18 Statistical analysis

The statistical analysis was performed on GraphPad prism (version 8.0) and data shown represented as a mean of at least *n* = 3 independent cell cultures preparations ±SEM. Shapiro–Wilk test of normality was performed; however, no outlier tests were performed. Statistical significance was assessed by repeated measures one‐way ANOVA, with sphericity assumed, followed by Tukey's post hoc test or by paired Student's t test where appropriate, unless stated otherwise. *p* < .05 were considered significant.

## 3 RESULTS

### 3.1 Overexpression of R47H TREM2 in mouse BV‐2 microglia increases phagocytosis of synaptosomes and neuronal loss

To determine the effects of TREM2 on microglial functions, wild‐type and R47H human TREM2 was stably expressed in a mouse BV‐2 microglial cell line. Endogenous mouse TREM2 had been knocked out by CRISPR/Cas9 in these cells as we were interested in the effects of human TREM2 variants, rather than mouse Trem2. These Trem2 knockout BV‐2 cells were transduced via lentivirus with plasmids expressing eGFP and (i) no TREM2 (KO Control), (ii) wild‐type human TREM2 (WT hTREM2), or (iii) R47H human TREM2 (R47H hTREM2). The three transduced cell lines were selected for expression of eGFP by flow cytometry. WT and R47H hTREM2‐expressing BV‐2 microglia expressed similar levels of human TREM2 mRNA to each other (Figure S1) and significantly more than KO Control. Their expression of human TREM2 mRNA was comparable to their expression of mouse Dap12 mRNA (Figure S1) and they expressed similar levels of TREM2 protein on the cell surface (Figure S2). WT and R47H hTREM2 expression slightly reduced numbers of BV‐2 cells cultured in 10% serum, and R47H TREM2 reduced cell numbers a bit more than WT hTREM2 (Figure S3). This reduction in cell numbers was probably due to a small decrease in proliferation, as cell death was very low (<2%).

**FIGURE 1:**
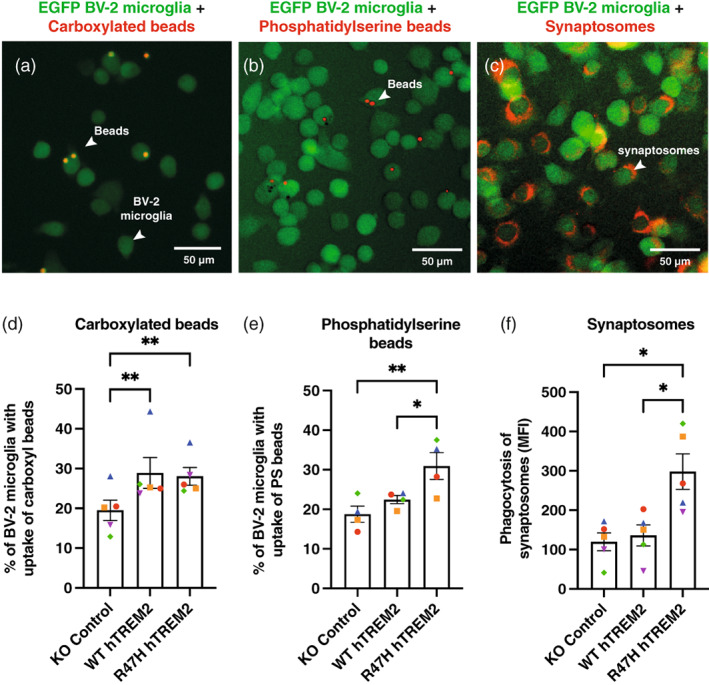
Overexpression of R47H hTREM2 increases BV‐2 phagocytic uptake of phosphatidylserine beads and synaptosomes compared with WT hTREM2 and TREM2 KO. (a, b, c) Microscopy images of BV‐2 microglia expressing eGFP (green) co‐cultured with (a) Carboxylated beads (red). (b) Rhodamine‐phosphatidylserine beads (red) and (c) pH‐rodo labeled synaptosomes (red). (d, e, f) Phagocytic uptake of three phagocytic targets was measured using flow cytometry. (d) Phagocytic uptake of sky‐blue 5 um carboxyl‐latex beads analyzed after 2 h co‐culture and shown as % uptake. (e) Rhodamine labeled phosphatidylserine beads analyzed after 1.5 h co‐culture shown as % uptake (f) synaptosomes isolated from rat and stained with pH‐rodo analyzed after 1.5 h co‐culture and shown as mean fluorescence intensity (MFI). (d, e, f) Cells were pre‐treated with the phagocytosis inhibitor cytochalasin D (CytoD) as a negative control. The CytoD negative control values have been removed from the data points presented. Each data point is the mean result from an independent experiment with three technical repeats. Co‐localization of phagocytic target in red with green BV‐2 cells shows target that has most likely been phagocytosed. Phagocytosis was quantified using flow‐cytometry and appropriate controls. Paired data points from the same experiment are represented by the same shape and color within a figure. Error bars are SEM and Tukey's multiple comparisons one‐way ANOVA was performed (d) KO Control versus WT hTREM2 **P = .0038, KO Control versus R47H hTREM2 **P = .0066 (e) KO Control versus R47H hTREM2 **P = .0066, WT hTREM2 versus R47H hTREM2 *P = .0333, KO Control versus WT hTREM2 ns = 0.3663 (f) KO Control versus R47H hTREM2 *P = .0127, WT hTREM2 versus R47H hTREM2 *P = .0209, KO Control versus WT hTREM2 ns = 0.9352.

We next investigated the effects of TREM2 expression on microglial phagocytosis using flow‐cytometry. WT and R47H hTREM2 expression in BV‐2 cells increased their phagocytosis of carboxylated beads, and the increase was similar for WT and R47H TREM2 expressing cells (Figure 1a,d). Because phosphatidylserine (PS) is a ligand for TREM2, we also tested phagocytosis of phosphatidylserine‐coated beads. R47H hTREM2 expressing BV‐2 cells phagocytosed significantly more phosphatidylserine‐coated beads than control cells, whereas WT hTREM2 expressing BV‐2 cells had no significant increase in phagocytosis of such beads relative to KO Control cells (Figure 1b,e). To use a more physiological target, we measured phagocytosis of pHrodo‐stained synaptosomes, that is, synapses, isolated from rat brain. WT hTREM2 expression did not affect phagocytosis of synaptosomes, but R47H hTREM2 expressing BV‐2 cells phagocytosed significantly more synaptosomes than both WT hTREM2 and KO control cells (Figure 1c,f). We checked for phosphatidylserine exposure on the surface of the synaptosomes using annexin V binding and found that most of the synaptosomes had exposed phosphatidylserine (Figure S4). Thus, R47H hTREM2 expression increased the phagocytosis of some targets relative to WT hTREM2 (synaptosomes and phosphatidylserine‐coated beads), but not others (carboxylated beads), suggesting the possibility that phosphatidylserine activates TREM2‐mediated phagocytosis more in R47H TREM2 expressing microglia than in WT TREM2 expressing microglia.

**FIGURE 2:**
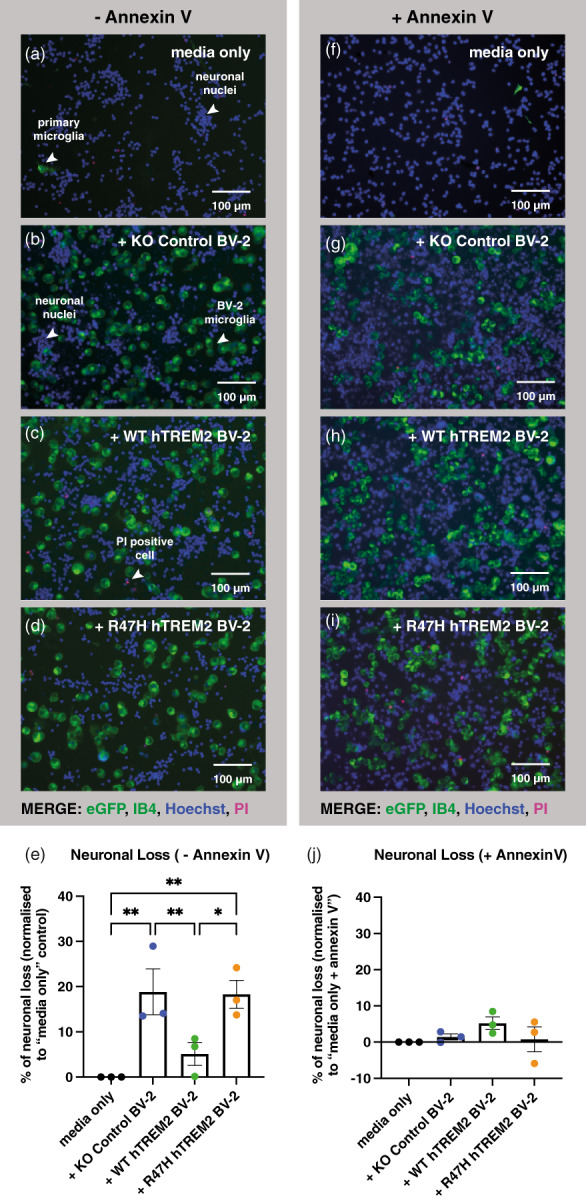
Co‐culturing primary cerebellar mouse neuronal‐glial co‐cultures with KO Control or R47H hTREM2 BV‐2 microglia induces significant neuronal loss after 24 h which is rescued by treatment with annexin‐V, an inhibitor of the eat‐me signal phosphatidylserine. Neuronal loss is not observed when co‐cultured with WT hTREM2 BV‐2 microglia. Primary neuronal‐glial co‐cultures, primarily consisting of neurons, were prepared from mouse cerebellum and seeded at 250,000 cells/well. 50,000 BV‐2 microglia (KO Control, WT hTREM2 or R47H hTREM2) or media control were added to these mixed primary neuronal‐glial cultures and left for 24 h ± 100 nM annexin V, an inhibitor of phosphatidylserine. After 24 h, cells were stained and imaged and density of microglia and of necrotic, apoptotic and healthy neurons was counted. (a–i) Representative images taken after 24 h of neuronal‐glial cerebellar cultures from mouse being (a, f) treated with media only control (b, g) co‐cultured with KO Control BV‐2 microglia (c, h) co‐cultured with WT hTREM2 BV‐2 microglia (d, i) co‐cultured with R47H hTREM2 BV‐2 microglia. (f–i) were treated with 100 nM annexin V immediately before addition of BV‐2 microglia. (a–i) Images show primary microglia (IB4, green), BV‐2 microglia (eGFP, green) necrotic cells (PI, red) and nuclei (Hoechst, blue) staining. Neurons were distinguished from astrocytes and microglia through their nuclear morphology and absence of IB4 staining. (e, j) Percentage of neuronal loss, which refers to loss of healthy neurons. The numbers of necrotic & apoptotic cells did not vary between conditions and were very low, less than 1% of cell population (data not shown). Data shown is from three biological repeats with each data point representing the mean result from one independent experiment. Each independent experiment was made up of three technical repeats and four 20x images were analyzed per technical repeat. Error bars are SEM and Tukey's multiple comparisons one‐way ANOVA was performed on raw, un‐normalized values for neuronal counts. “Media only” versus “+ KO Control BV‐2” **P = .0070, “media only” versus “+ R47H hTREM2 BV‐2” **P = .0072, “+ KO Control BV‐2” versus “+ WT hTREM2 BV‐2” *P = .0396, “+ WT hTREM2 BV‐2” versus “+ R47H hTREM2 BV‐2” *P = .0414.

In order to test whether TREM2 expression affects neuronal loss, we added BV‐2 expressing WT or R47H hTREM2 or KO Control BV‐2 (not expressing TREM2) or a media only control to primary neuronal‐glial cultures from mice, and 24 h later counted the number of live neurons (Figure 2). Control BV‐2 cells and R47H‐expressing BV‐2 cells induced loss of about 20% of the neurons in these co‐cultures, whereas WT hTREM2‐expressing BV‐2 cells induced no significant neuronal loss (Figure 2e). This suggests that expression of WT TREM2 inhibits the neuronal loss induced by BV‐2 cells lacking TREM2, whereas R47H TREM2 expressing BV‐2 cells increase neuronal loss relative to WT TREM2 expressing BV‐2 cells. Note, however, that neuronal loss is equally induced by BV‐2 cells expressing R47H TREM2 and those lacking TREM2. Neurons were distinguished from glial cells based on their morphology and absence of Isolectin B4 (IB4) staining (Figure S5), and validated using the live neuronal marker NeuO (Figure S6). Necrotic cells (staining with propidium iodide, PI) and apoptotic cells (condensed nuclei) were <1% of cells (Figure 2).

We have previously found that microglia can phagocytose co‐cultured neurons if stressed neurons expose the eat‐me signal phosphatidylserine, and this can be prevented by adding annexin V to block any exposed phosphatidylserine on the neuronal surface (Neher et al., 2011; Neniskyte et al., 2011). Therefore, we tested whether the neuronal loss induced by adding BV‐2 cells could be blocked by annexin V. Addition of 100 nM annexin V fully prevented the neuronal loss induced by addition of all types of BV‐2 cells (Figure 2f–j). This suggests that the neuronal loss induced by addition of BV‐2 cells is mediated by phosphatidylserine exposure on neurons, both in the presence and absence of TREM2.

### 3.2 Overexpression of R47H TREM2 in human CHME‐3 microglia increases phagocytosis of synaptosomes and neuronal loss

To test the effects of TREM2 in human cells, wild‐type and R47H TREM2 (plus eGFP) were stably expressed in the human CHME‐3 microglial cell line (also known as HMC3 cells). Before transduction, these cells expressed DAP‐12 but little endogenous TREM2, and after lentiviral transduction the wild‐type TREM2 (WT TREM2) or R47H TREM2 (R47H TREM2) expressing cells expressed similar levels of TREM2 mRNA and cell surface protein, although R47H TREM2 expression was somewhat lower (Figure S7). Control cells were transduced with vector expressing eGFP only, but no TREM2. Expression of WT TREM2 increased cell attachment to laminin and fibronectin, relative to control cells (Figure S8a,b), and mildly increased proliferation relative to control cells (Figure S9). Expression of R47H TREM2 did not increase cell attachment to laminin and fibronectin (Figure S8a,b), and mildly decreased proliferation relative to control cells (Figure S9).

**FIGURE 3:**
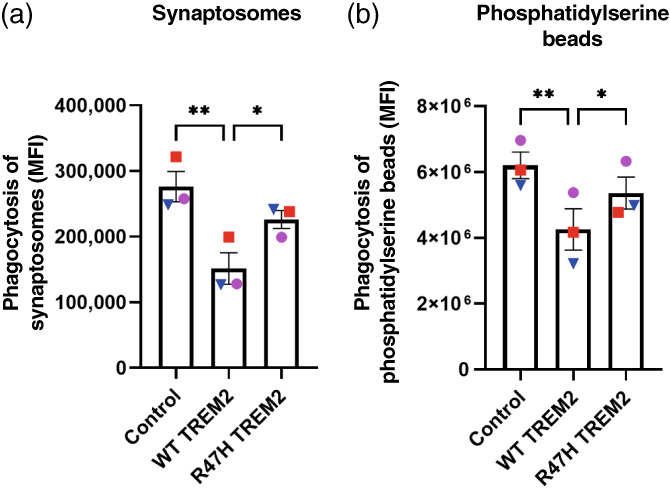
Overexpression of WT TREM2 in CHME‐3 cells reduces phagocytosis of synaptosomes and phosphatidylserine coated beads. Phagocytosis of (a) Synaptosomes isolated from rat and stained with pH‐rodo analyzed after 1 h of treatment and shown as mean fluorescence intensity (MFI) and (B) rhodamine‐labeled phosphatidylserine‐coated beads analyzed after 1 h of treatment and shown as MFI. Overexpression of WT TREM2 decreased uptake of both targets compared to control. R47H expression also decreased uptake of synaptosomes and phosphatidylserine‐coated beads but did so less than WT TREM2. Repeats done on the same day with the different cell lines are depicted by the same color and symbols. (a & b) *N* = 3, statistics: repeated measures, one way ANOVA, followed by Tukey's post hoc test.*p < .05, **p < .01.

To test the effect of TREM2 variants on phagocytosis, we incubated TREM2 expressing CHME‐3 cells with two different phagocytic targets: (A) rat synaptosomes or (B) phosphatidylserine‐coated beads. Expression of WT TREM2 reduced phagocytosis of both targets relative to control cells (Figure 3a,b). Expression of R47H TREM2 increased phagocytosis of synaptosomes and phosphatidylserine‐coated beads relative to WT TREM2 expressing cells (Figure 3a–c). This indicates that WT TREM2 expression reduces non‐specific phagocytosis in CHME‐3 cells, whereas R47H TREM2 has a target specific effect, which as in BV‐2 cells increases phagocytosis of synaptosomes and phosphatidylserine‐coated beads relative to WT TREM2.

**FIGURE 4:**
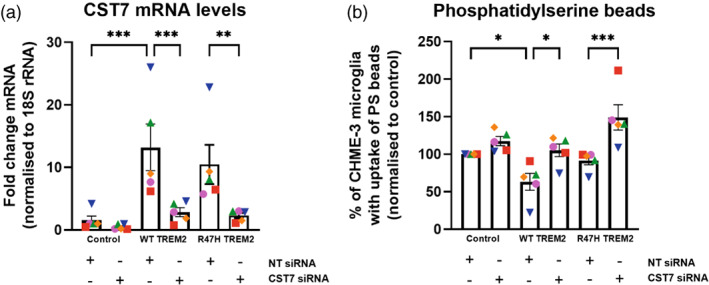
TREM2 induces an increase in *CST7* mRNA expression and knock down of *CST7* increases phosphatidylserine coated bead uptake in TREM2 overexpressing CHME‐3 cells. (a) Data is expressed as fold change over non‐target control and all samples were normalized to 18 S rRNA housekeeping gene. *CST7* mRNA level is increased when TREM2 is present and when treated with RNAi against *CST7*, *CST7* is successfully reduced in all cell lines by at least 50%. RNA was extracted from CHME‐3 microglia 48 h after treatment with non‐target (scrambled) or *CST7* targeting RNAi. *N* = 5. Repeats done on the same day with the three variant cell lines are depicted by the same color and symbols. Statistics: repeated measures, one way ANOVA with Tukey's post hoc test. **p < .01, ***p < .001. (b) Uptake of rhodamine‐labeled, phosphatidylserine‐coated 3 micron beads by control, WT and R47H TREM2 overexpressing CHME‐3 cells 48 h after treatment with non‐target (scrambled) or *CST7* targeting RNAi. Phagocytosis was analyzed after 1 h treatment of CHME‐3 cells with phosphatidylserine‐coated beads. *N* = 5. Statistics: repeated measures, one way ANOVA with Tukey's post hoc test. * *p* < .05, *** *p* < .001.

To try and understand the changes in phagocytosis induced by TREM2 expression, we determined the expression of Disease Associated Microglia (DAM) genes. We found that expression of either WT or R47H TREM2 had the tendency to reduce the expression of *P2RY12* and *TMEM119*, though this was not statistically significant (Figure S10). However, expression of either WT or R47H TREM2 did substantially increase the expression of *CST7* (Figure S10)*. CST7* encodes cystatin F, an inhibitor of lysosomal cathepsins and microglial phagocytosis (Kang et al., 2018; Kos et al., 2018; Ofengeim et al., 2017), which is highly upregulated by TREM2 (Keren‐Shaul et al., 2017). Consequently, we tested whether knockdown of *CST7* expression reversed the TREM2‐induced inhibition of phagocytosis by comparing the phagocytosis of phosphatidylserine‐coated beads by the CHME‐3 cells treated with a siRNA targeting *CST7* or non‐target siRNA (Figure 4). The siRNA strongly reduced the increase in *CST7* expression induced by TREM2 expression (Figure 4a). As expected, knockdown of *CST7* had no effect on phagocytosis by control cells (Figure 4b), which express minimal *CST7* (Figure 4a). However, knockdown of *CST7* in WT TREM2 expressing cells increased phagocytosis to the control level, that is, knockdown of *CST7* prevented WT TREM2 expression from inhibiting phagocytosis (Figure 4b). Knockdown of *CST7* also increased phagocytosis of phosphatidylserine‐coated beads in the R47H TREM2 expressing cells (Figure 4b). This suggest that there are two effects of TREM2 expression: (i) an inhibition of general phagocytosis due to *CST7/*cystatin F induction in both WT and R47H expressing cells, and (ii) an increase in phosphatidylserine‐specific phagocytosis particularly in the R47H TREM2 expressing cells.

**FIGURE 5:**
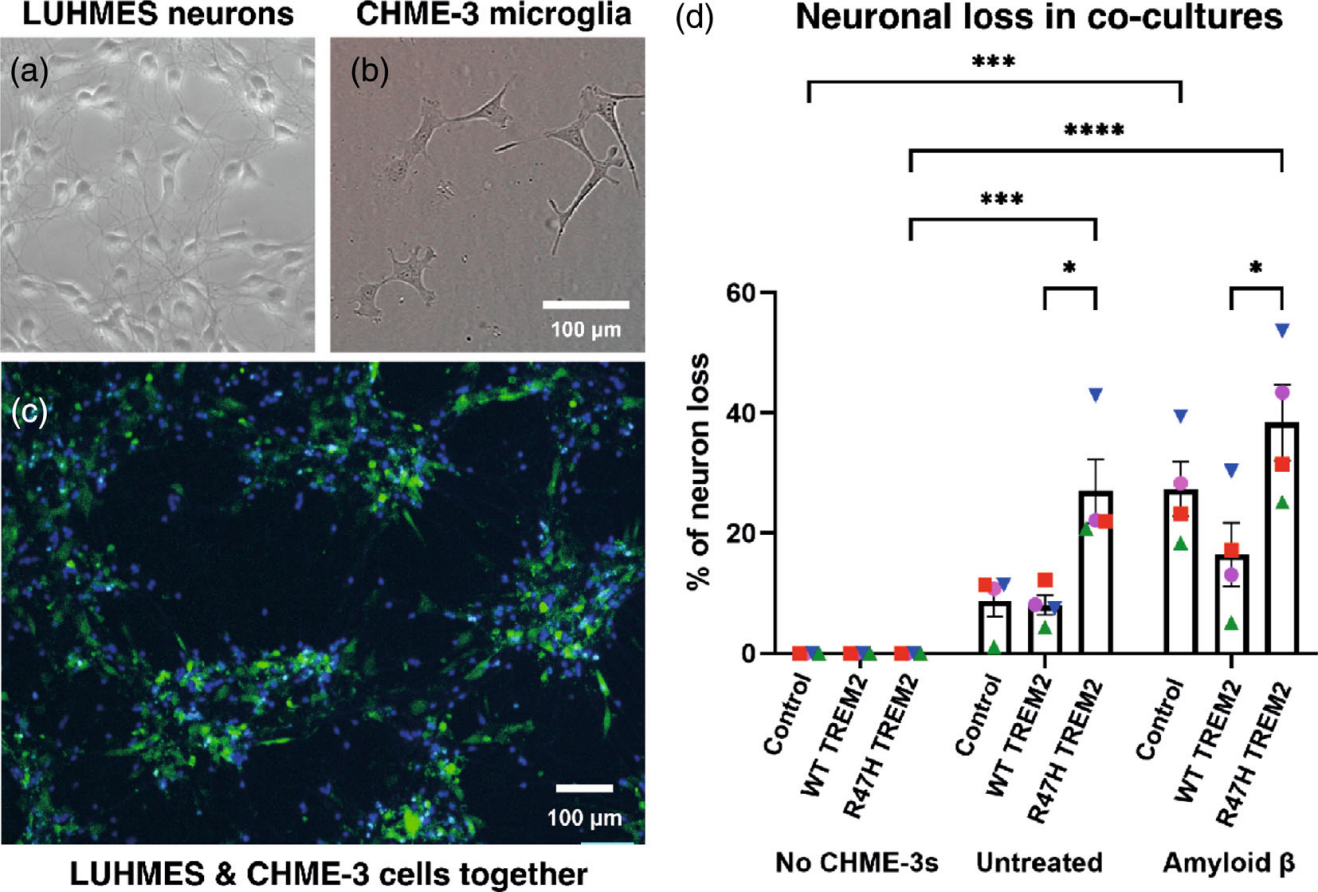
Overexpression of R47H TREM2 mutant in CHME‐3 microglia leads to increased neuron (LUHMES) loss in co‐culture with neurons. (a) Image of differentiated LUHMES cells. (b) Image of CHME‐3 cells. (c) Image of LUHMES (blue) and CHME‐3 (green) co‐culture. (d) Percentage neuron loss in LUHMES:CHME‐3 (100,000: 100,000) co‐culture after treatment ±2 μM Aβ for 48 h. Percentage loss is compared to neuron only control. Number of neurons was identified via blue CellTrace Violet staining and counted using automated software. *N* = 4. Statistics: two‐way ANOVA, with Sidak's post hoc test. *p < .05, ***p < .001, ****p < .0001.

We then investigated whether TREM2 expressing CHME‐3 cells would induce neuronal loss by co‐culturing CHME‐3 cells with LUHMES (Lund Human Mesencephalic neuronal) cells that had been differentiated into human neuron‐like cells, and treating ±2 μM Aβ for 48 h (Figure 5a–c). In the absence of Aβ, only the R47H expressing CHME‐3 cells induced significant loss of LUHMES neurons (Figure 5d). In the presence of Aβ, there was neuronal loss with all three CHME‐3 lines, but only the control and R47H TREM2 expressing CHME‐3 cells induced a statistically significant level of neuronal loss, and the R47H expressing cells induced significantly more neuronal loss than the WT expressing cells (Figure 5d). Thus, R47H TREM2 expression can increase neuronal loss.

### 3.3 R47H TREM2‐expressing human iPS‐Mg have increased phagocytosis of synaptosomes

**FIGURE 6:**
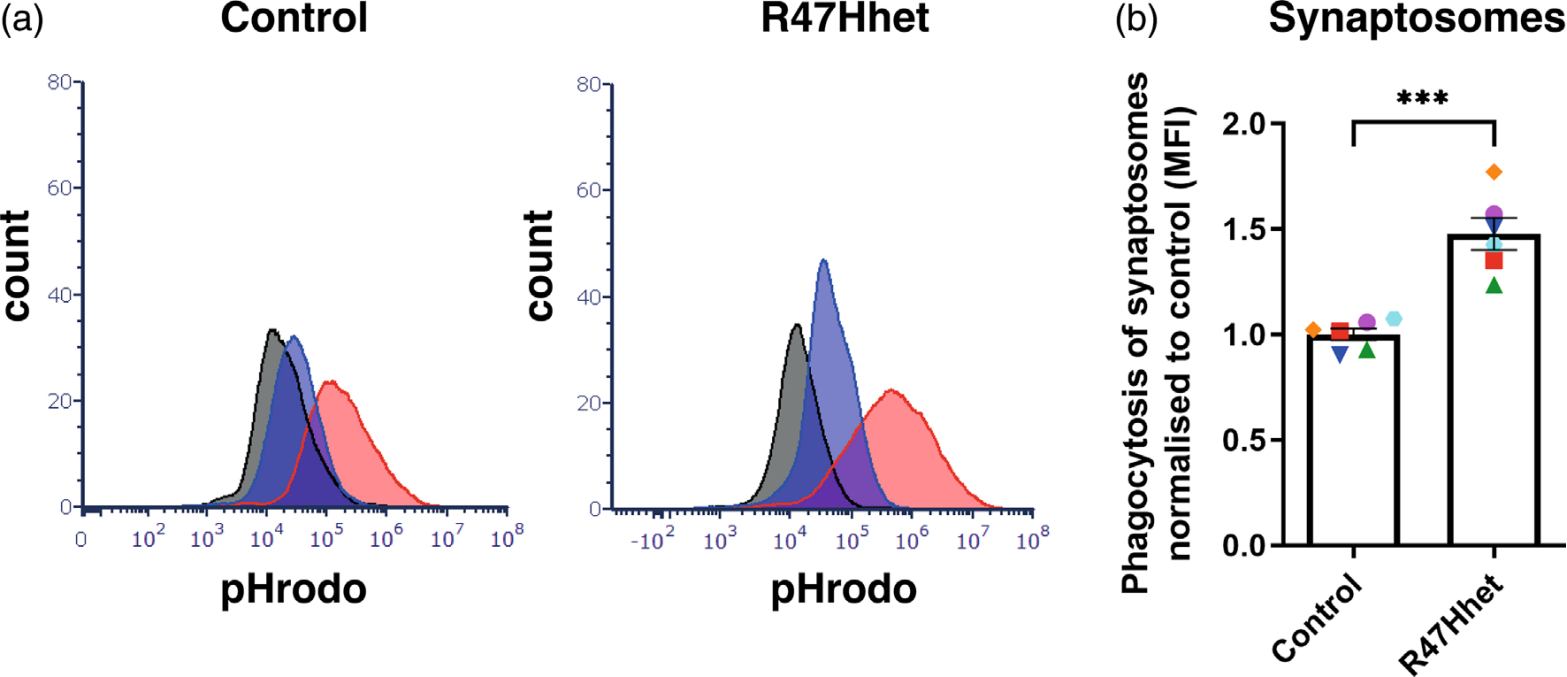
Heterozygous R47H expression in microglia‐like iPSCs results in increased uptake of rat cortical synaptosomes. (a) Uptake of pH‐Rodo stained synaptosomes by control (BIONi010‐C) and heterozygous R47H (ADRC8.6 and 26.15) microglia‐like hiPSCs. R47H heterozygous derived hiPSCs increased uptake of synaptosomes compared to control line. Data is normalized to mean of control and has had cytochalasin D control subtracted to show active phagocytosis. *N* = 6 of control (BIONi010‐C), from two different differentiations and *N* = 3 from both ADRC8.6 and 26.15 (R47Hhet clones). Repeats done on the same day with the different cell lines are depicted by the same color and symbols. Statistics: unpaired student's *t*‐test. ***p < .001. (b) Representative flow plots of pH‐Rodo stained synaptosomes by control and R47H heterozygous microglia‐like hiPSCs. Black histogram = untreated cells (no cargo), red histogram = cells with cargo (Ph‐Rodo stained synaptosomes) and blue histogram = cells with cargo +1 h pre‐treatment with 10uM cytochalasin D.

As BV‐2s and CHME‐3s are cell lines, we sought to test the relative effects of WT and R47H TREM2 expression on phagocytosis of synaptosomes by human induced pluripotent stem cells differentiated into microglia‐like cells (iPS‐Mg). We found that iPS‐Mg derived from heterozygous R47H TREM2 individuals (ADRC8.6 and 26.15) had higher phagocytosis of synaptosomes than iPS‐Mg from controls (expressing common variant/wild‐type TREM2, BIONi010‐C) (Figure 6a). Thus, endogenous expression of R47H TREM2 in iPS‐Mg increases phagocytosis of isolated synapses, just as exogenous expression of R47H TREM2 does in BV‐2 and CHME‐3 cells.

### 3.4 R47H TREM2‐expressing human iPSC‐derived microglia have increased TREM2 activation of SYK by phosphatidylserine liposomes

**FIGURE 7:**
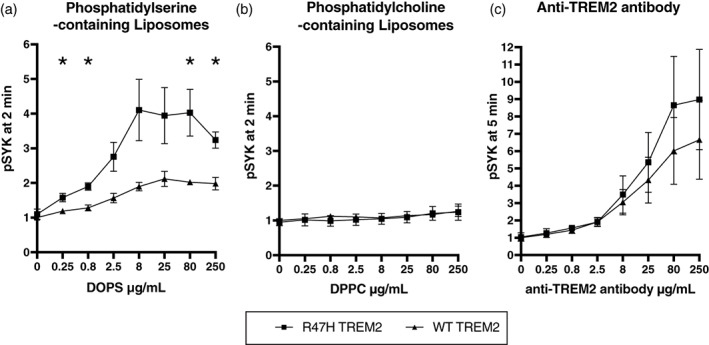
pSYK activation by liposomes & antibodies in hiPSC‐derived microglia. hiPSC‐derived microglia expressing homozygous wild‐type or R47H TREM2 were exposed to the indicated concentrations of (a) phosphatidylserine‐containing liposomes, (b) phosphatidylserine‐containing liposomes or (c) anti‐TREM2 antibodies, and phosphorylated SYK (pSYK) measured by alphaLISA, 2 min after adding liposomes or 5 min after adding antibodies. pSYK is normalized to the level before addition of liposomes or antibodies. The experiment was repeated on 3 separate occasions, of which means and SEM are represented. Significant differences between WT and R47H TREM2 expressing cells were tested by unpaired t test at each concentration, *p < .05.

As R47H‐expressing iPS‐Mg had increased uptake of synaptosomes, we tested whether R47H TREM2 expression affected activation of TREM2 by phosphatidylserine. Common variant/WT TREM2 and homozygous R47H TREM2‐expressing iPS‐Mg were activated with phosphatidylserine liposomes, containing a physiological mixture of cell membrane lipids (Boudesco et al., 2022), and the level of phospho‐SYK was measured by AlphaLISA. SYK tyrosine kinase is immediately downstream of TREM2 and mediates TREM2‐induced phagocytosis (Olufunmilayo & Holsinger, 2022). Phosphatidylserine‐containing liposomes caused a 3‐fold greater activation of SYK in the R47H TREM2‐expressing iPS‐Mg than in the common variant/WT TREM2‐expressing iPS‐Mg (Figure 7a). Liposomes not containing phosphatidylserine did not cause any significant activation of SYK in either cell line (Figure 7b). Anti‐TREM2 antibodies caused activation of SYK that was not significantly different between common variant/WT and homozygous R47H TREM2 expressing iPS‐Mg, although there was a trend for increased activation of the R47H TREM2 expressing cells (Figure 7c). WT and R47H TREM2 iPS‐Mg cells also had the same pSYK response to concanavalin A, which activates pSYK independent of TREM2 (Figure S11). The increased response of R47H TREM2 expressing cells was not due to greater expression, as these cells actually expressed less TREM2 than WT TREM2 expressing cells (Figure S11), as has been reported previously (Hall‐Roberts et al., 2020). Thus, phosphatidylserine (within a physiological mixture of phospholipids) appears to activate R47H TREM2 more than common variant/WT TREM2. And this is consistent with R47H TREM2 expressing cells phagocytosing more phosphatidylserine‐exposing targets than WT TREM2 expressing cells, as shown in Figures 1, 3 and 6.

## 4 DISCUSSION

We found that for both BV‐2 and CHME‐3 cells, R47H TREM2 expressing cells phagocytosed more phosphatidylserine‐coated beads than WT TREM2 expressing cells, consistent with greater activation of TREM2 by phosphatidylserine in R47H TREM2 expressing cells. In BV‐2, CHME‐3 and iPS‐Mg, the R47H TREM2 expressing cells phagocytosed more synaptosomes. This is consistent with the finding of Hall‐Roberts et al. (2020) that R47H TREM2 expressing hiPSC‐derived macrophages appeared to have phagocytosed more synaptosomes than WT TREM2 expressing cells.

For both BV‐2 and CHME‐3 cells, R47H TREM2 expressing cells caused more neuronal loss than WT TREM2 expressing cells, and the neuronal loss induced by BV‐2 cells was prevented by blocking phosphatidylserine with annexin V. We have previously shown that microglia can induce neuronal loss by phagocytosing stressed‐but‐viable neurons, reversibly exposing phosphatidylserine, and this can be prevented by blocking phosphatidylserine with annexin V (Neher et al., 2011; Neniskyte et al., 2011). These findings immediately suggest an explanation of why people heterozygous for R47H TREM2 have a four‐fold increased risk of AD, that is, R47H TREM2 causes increased microglial phagocytosis of synapses and neurons via exposed phosphatidylserine. Note that we did not show here that the neuronal loss was by phagocytosis, but have shown this previously (Neher et al., 2011; Neniskyte et al., 2011). And, using flow‐cytometry, we measured microglial phagocytosis of pHrodo‐labeled synaptosomes by the fluorescence induced when these are taken into microglial lysosomes, rather than directly imaging synaptosomes within the lysosomes.

Our finding that phosphatidylserine‐containing liposomes activate R47H TREM2 signaling more than WT TREM2 signaling may provide an explanation of why R47H TREM2 increases phagocytosis of phosphatidylserine‐specific targets, relative to WT TREM2. Previous reports of the response of R47H TREM2 to phosphatidylserine are somewhat contradictory. For example, Wang et al. (2015) and Song et al. (2017) constructed TREM2‐expressing reporter cells, expressing GFP under an NFAT promotor, activated overnight on lipid coated plates, and found a greatly reduced response in R47H TREM2 expressing cells to multiple phospholipids, including phosphatidylserine. However, surprisingly, they found the strongest and most sensitive TREM2 response was to phosphatidylcholine, and this was the same in WT and R47H TREM2 expressing cells. In contrast, Sudom et al. (2018) showed poor binding of both WT and R47H TREM2 ectodomain to phosphatidylcholine. They found reduced pSYK activation by phosphatidylserine liposomes in HEK cells expressing R47H relative to WT TREM2, and found that the R47H TREM2 ectodomain bound less to phosphatidylserine‐coated plates, although no statistics were shown. By contrast, Kober et al. (2016) used TREM2 ectodomain and found equal binding of WT and R47H TREM2 to phospholipids, including phosphatidylserine, as liposomes or coated on plates in pure form. Hall‐Roberts et al. (2020) used hiPSC‐derived macrophages, and found no difference in the pSYK response between WT and R47H TREM2 to dead neurons. Cosker et al. (2021) used iPS‐Mg and reported that heterozygous or homozygous R47H TREM2 prevented a pSYK response to phosphatidylserine liposomes. These previous reports have used pure phosphatidylserine, rather than a physiological mix of phospholipids as here, and it is possible that this affects TREM2 binding. For example, the binding of TREM2 to pure phosphatidylserine might be dominated by the charge interaction between the very negative liposomes and the positively‐charged ligand‐binding surface of TREM2, which is disrupted by R47H (Sudom et al., 2018). Whereas when a relatively low phosphatidylserine concentration is distributed among a physiological mix of neutral phospholipids and cholesterol, then the specific binding of TREM2 might be enhanced. This is supported by the recent report that these physiological liposomes are better ligands for TREM2 and that R47H TREM2 is preferentially activated by phosphatidylserine in these liposomes (Boudesco et al., 2022).

In CHME‐3 cells, WT TREM2 expression inhibited the phagocytosis of some targets, and this appeared to be due to the induced expression of cystatin F. Cystatin F, which is expressed from the *CST7* gene, is part of the Disease Associated Microglia (DAM) gene profile known to be induced by TREM2 (Keren‐Shaul et al., 2017) and overexpressed in AD brains (Keren‐Shaul et al., 2017; Ofengeim et al., 2017). However, we still do not know whether the DAM phenotype is beneficial or detrimental in disease. Cystatin F is known to inhibit microglial phagocytosis (Kang et al., 2018; Ofengeim et al., 2017), and we showed that the inhibition of phagocytosis induced by TREM2 expression is mediated by cystatin F induction. Thus, cystatin F may be repressing excessive phagocytosis in disease associated microglia. We do not know why expression of WT TREM2 in CHME‐3 cells reduced phagocytosis of synaptosomes or phosphatidylserine‐coated beads, but not in BV‐2 cells, but clearly it might be because BV‐2 cells are mouse cells and CHME‐3 are human cells, with a different transcriptional response to TREM2 expression. Expression of WT TREM2 in BV‐2 or CHME‐3 reduced neuronal loss, relative to the TREM2‐lacking control cells, and this is consistent with WT TREM2 inhibiting phagocytosis of the neurons by general down‐regulation of phagocytosis via induction of cystatin F. Thus, TREM2 appears to have two opposing effects on the phagocytosis of phosphatidylserine‐expressing targets: a stimulation due to TREM2 acting as a phagocytic receptor for these targets, and an inhibition due to the induced expression of CST7. R47H TREM2 appears to have a greater stimulation by phosphatidylserine‐expressing targets, but the same induction of CST7 as for WT TREM2, and therefore there is increased phagocytosis of phosphatidylserine‐expressing targets by R47H TREM2 expressing microglia.

In the literature, there are mixed results on the effect of wild‐type and R47H TREM2 on phagocytosis. In cultured microglia, Trem2 knockout has been found to reduce phagocytosis of synaptosomes after 24 h of incubation, but with no difference after 2 h (Filipello et al., 2018; McQuade et al., 2020). Primary microglia from Trem2 knockout mice had no change in phagocytosis of beads or liposomes containing 50% phosphatidylserine, but decreased phagocytosis of 99% phosphatidylserine liposomes (Scott‐Hewitt et al., 2020). Knockdown of Trem2 in BV‐2 cells reduced phagocytosis of injured/dead neurons (Kawabori et al., 2015). Whereas Schoch et al. (2021) found that Trem2 knockdown in cultured microglia increased phagocytosis. In vivo, in different rodent models of disease, loss of wild‐type mTREM2 has been associated with either decreased (Wang et al., 2015) or increased phagocytosis (Schoch et al., 2021), and either decreased (Leyns et al., 2017) or increased synaptic and/or neuronal loss (Lee et al., 2021; Wang et al., 2015). R47H TREM2 knock‐in mouse models have been associated with loss of function (Cheng‐Hathaway et al., 2018). However, Xiang et al. (2018) found that R47H knock‐in induced a cryptic splice site in mice, resulting in reduced expression of R47H, making the R47H knock‐in equivalent a TREM2 knockout. Interestingly, this splicing only occurred in mice and not in microglia‐like R47H TREM2 iPS‐Mg (Xiang et al., 2018). In AD and MCI patients, the heterozygous R47H TREM2 variant was associated with substantially increased loss of cortical gray matter (Luis et al., 2014). More recently, Sayed et al. (2021) found that microglia from AD patients with R47H TREM2 were hyperactivated consistent with increased TREM2 signaling, and they generated mice expressing heterozygous R47H and found this exacerbated tauopathy, consistent with the toxic gain‐of‐function of R47H found here.

We did not compare the effects of WT and R47H TREM2 on the general expression profile of microglia, but Sayed et al. (2021) found that human and mouse microglia expressing heterozygous R47H TREM2 had increased expression of DAM genes, suggesting increased signaling. Similarly, Korvatska et al. (2020) found that heterozygous R47H expression in AD patient brains upregulated expression of type I response and pro‐inflammatory cytokine genes. Ellwanger et al. (2021) found that R47H TREM2 versus WT TREM2 expression had little effect on microglial expression states in 5xFAD mice, but reduced the response to an anti‐TREM2 antibody in some conditions. Hall‐Roberts et al. (2020) found that R47H expression changed hiPSC‐derived microglial expression of multiple genes, but had only subtle effects on microglial functions, including increased phagocytosis of synaptosomes.

## 5 CONCLUSIONS

In this study, we have identified novel roles of WT and R47H TREM2 in phagocytosis of synapses and neurons (Figure S12), which are consistent with emerging findings. However further work is needed to understand the exact molecular mechanisms by which R47H TREM2 gains function and how this may contribute to neurodegenerative disease.

## AUTHOR CONTRIBUTIONS

Alma S. Popescu, Claire A. Butler, Thomas M. Piers, and Alessandro Cinti performed the experiments. Alma S. Popescu, Claire A. Butler, David H. Allendorf, Anna Mallach, Julian Roewe, Peter Reinhardt, Loredana Redaelli., Christophe Boudesco, Laurent Pradier, Jennifer M. Pocock, and Peter Thornton designed, produced, or provided the cell systems used in these experiments. Guy C. Brown, Alma S. Popescu, and Claire A. Butler conceptualized the majority of the research. Guy C. Brown supervised the research. Guy C. Brown, Alma S. Popescu& Claire A. Butler wrote the majority of the manuscript. All authors reviewed and approved the manuscript.

## FUNDING INFORMATION

This project has received funding from the Innovative Medicines Initiative 2 Joint Undertaking under grant agreement No 115976. This Joint Undertaking receives support from the European Union's Horizon 2020 Research and Innovation Programme and EFPIA. This project was also funded by the Biotechnology and Biological Sciences Research Council (BBSRC, BB/R506047/1).

## CONFLICT OF INTEREST

The authors declare no conflicts of interest.

## Supporting information


**FIGURE S1.** TREM2 & DAP12 expression in BV‐2 cells. Stable BV‐2 microglia cell‐lines were created using lentiviral transduction with plasmids containing gene of interest and eGFP. (a, b, c) RT‐qPCR using RNA from mTREM2 KO BV‐2 microglia transduced with either control vector (KO), hTREM2 WT (WT) or hTREM2 R47H (R47H). (a) RT‐qPCR amplification curves showing expression of *mGapdh* (blue), *mDap12* (green) and *hTREM2* (orange). Take‐off points for *hTREM2* and *mDap12* are comparable for hTREM2 WT and hTREM2 R47H BV‐2 cells. (b, c) Expression is normalized to the geometric mean of housekeepers *Gapdh* & *Gusb* and fold changes are shown relative to KO Control BV‐2. Error bars represent SEM, ordinary one‐way ANNOVA performed on raw comparative concentration values not normalized to KO Control BV‐2 data. Brown‐Forsythe and Welch post hoc test applied. KO Control BV‐2 versus WT hTREM2 BV‐2 **P = .0027, KO Control BV‐2 versus R47H hTREM2 BV‐2 **P = .0076

**FIGURE S2.** Expression of TREM2 protein on surface of BV‐2 microglia cell‐lines. (a) MFI (PE) values for KO Control BV‐2, WT hTREM2 BV‐2 & R47H hTREM2 BV‐2 using rat anti‐human/mouse TREM2 antibody (clone: 237920) with corresponding MFI values for rat IgG2B isotype control antibody (clone: 141945) subtracted (*N* = 3). Error bars represent S.E.M, paired one‐way ANNOVA performed, followed by Tukey's post hoc test. KO Control BV‐2 versus WT hTREM2 BV‐2 *P = .0468, KO Control BV‐2 versus R47H hTREM2 BV‐2 *P = .0276. (b) Representative histograms of TREM2 protein expression on the surface of the three BV‐2 cell lines; KO Control BV‐2, WT hTREM2 BV‐2 & R47H hTREM2 BV‐2. Histogram for isotype control antibody shown in red and rat anti‐human/mouse TREM2 shown in blue. Both antibodies are conjugated to phycoerythrin (PE) and so a shift in the PE channel represents increased TREM2 expression on surface of microglia.

**FIGURE S3.** TREM2 expression mildly reduces proliferation. Overexpression of TREM2 appears to slightly decrease BV‐2 microglia proliferation after 24 h, with R47H TREM2 significantly decreasing proliferation versus KO Control BV‐2 cells. 500,000 BV‐2 cells were seeded at in DMEM supplemented with 10% heat‐inactivated FBS and 1% Pen/Strep for 24 h after which proliferation was measured by cell density (counted using hemocytometer). *N* = 3. Data points with the same color/shape represent paired data carried out in the same experimental repeat. Error bars represent SEM, paired one‐way ANNOVA performed, followed by Tukey's post hoc test. KO Control BV‐2 versus R47H hTREM2 BV‐2 *P = .0249

**FIGURE S4.** Phosphatidylserine exposure on synaptosomes shown using PE/Dazzle™ conjugated Annexin‐V. Synaptosomes were combined with PE/Dazzle™‐annexin V which binds to exposed phosphatidylserine. Synaptosomes were then analyzed via flow‐cytometry. Shift of red histogram (synaptosomes labeled with PE/Dazzle™ annexin V) versus blue histogram (synaptosomes without labeling) in the PE/Dazzle™ channel represents population of synaptosomes exposing phosphatidylserine.

**FIGURE S5.** Representative phase and merged images of primary cerebellar mouse neuronal‐glial cultures co‐cultured with KO Control, WT hTREM2 BV‐2 or R47H hTREM2 BV‐2 microglia ± annexin‐V for 24 h. Phase images for each condition are shown alongside their corresponding composite image of the phase channel and three fluorescent channels; red (PI‐positive necrotic cells), green (IB4‐positive microglia and eGFP expressing BV‐2 microglia) and blue (nuclei stained with Hoechst). Neurons were distinguished from astrocytes and microglia through their nuclear morphology and absence of IB4 staining.

**FIGURE S6.** Primary cerebellar mouse neuronal‐glial cultures stained with live neuronal marker. Cultures were stained with a marker of live‐neurons NeuO (green), a microglial marker IB4 (red) and nuclear marker Hoechst (blue). These images are shown alongside a composite image of the three fluorescence channels and alongside the corresponding phase image. NeuO was used to validate the used method of distinguishing nuclei using nuclear morphology and an absence of IB4 staining.

**FIGURE S7.** TREM2 & DAP12 expression in CHME‐3 cells at mRNA level and expression of TREM2 protein on CHME‐3 cell surface. qPCR analysis of CHME‐3 cells expressing (a) human TREM2 and (b) human DAP12. (c) Human TREM2 protein expression on cell surface of CHME‐3 cells. (d–f) flow plots of CHME‐3 cells expressing TREM2 in (d) in control cell line (EGFP only), (e) WT TREM2 overexpressing cell line, and (f) R47H TREM2 overexpressing cell line. Data shown are for one biological repeat. Red histogram = monoclonal rat IgG2B isotype control antibody (clone: 141945), blue histogram = rat anti‐human/mouse TREM2 (clone: 237920). All antibodies were conjugated to phycoerythrin (PE).

**FIGURE S8.** Overexpression of TREM2 (WT and R47H mutant) modulates attachment to laminin and fibronectin and proliferation. (a) WT TREM2 increases attachment to 20 μg/ml laminin over 4 h (b) WT TREM2 increases attachment to 10 μg/ml fibronectin over 240 min. Attachment was measured by electrical impedance of cell layer. *N* = 3. Statistics: repeated measures one way ANOVA, followed by Tukey's post hoc test, on end point (240 min) value. * is compared to control. *p < .05.

**FIGURE S9.** TREM2 expression mildly modulates proliferation in CHME‐3 cells. WT TREM2 increases, while R47H mutant decreases, proliferation of CHME‐3 cells after seeding the same density of cells in presence of DMEM supplemented with 10% heat‐inactivated FBS and 1% antibiotics for 48 h. Proliferation measured by cell density (counted using hemocytometer). *N* = 4. Repeats done on the same day with the three variant cell lines are depicted by the same color and symbols. Statistics: repeated one‐way ANOVA, followed by Tukey's post hoc test. *p < .05, **p < .01.

**FIGURE S10.** *CST7* mRNA expression is significantly increased in WT and R47H TREM2 overexpressing CHME‐3 cells. qPCR analysis of key microglial genes indicative of disease associated microglia (DAM). *CST7* is significantly upregulated in TREM2 overexpressing CHME‐3 cells. *CD33*, *TMEM119*, *P2RY12* and *CX3CR1* mRNA levels are not significantly changed between the different cell lines. However, there is a trend toward reduced levels of homeostatic markers *TMEM119*, *P2RY12* and *CX3CR1*. Data are expressed as fold change over the control (EGFP only) cell line and all samples are normalized to 18 S rRNA housekeeping gene. *N* = 3. Repeats done on the same day with the three variant cell lines are depicted by the same color and symbols. Statistics: ratio *t*‐test comparing control to WT TREM2 or R47H TREM2 separately. *p < .05, **p < .01.

**FIGURE S11.** Comparison of (a) number of cells, (b) pSYK activation by concanavalin A, and (c) amount of sTREM2 and total TREM2, in wild‐type and R47H TREM2 expressing hiPSC‐derived microglia. (a) iPSC‐Mg were seeded and maturated in 384‐well plate. Homogeneity of cell seeding was confirmed by counting nuclei for both WT and R47H at the end of maturation. Data shown represent mean ± *SD*. (b) Comparable TREM2‐independent activation of SYK is detectable in both WT and TREM2‐R47H IPSC‐Mg after treatment with 500 ng/ml Concanavalin A. Data shown represent mean ± *SD*. (c) Soluble TREM2 (sTREM2) and cellular TREM2 (TOT TREM2) were quantified by Homogeneous Time Resolved Fluorescence (HTRF) using anti‐TREM2 antibodies to probe the cellular supernatant or cell lysates.

**FIGURE S12.** Summary of findings and potential means by which R47H TREM2 potentiates Alzheimer's disease. Activation of TREM2 on microglia may have two opposing effects on the phagocytosis of synaptosomes and other phosphatidylserine‐exposing targets: (i) a stimulation due to TREM2 acting as a phagocytic receptor for these targets, and (ii) an inhibition due to the induced expression of *CST7*. Which of these opposing effects predominates is likely to depend on conditions such as time course, phosphatidylserine exposure and TREM2 variant. Stimulation of both WT and R47H TREM2 on microglia induces expression of *CST7*, a disease associated microglia (DAM) gene that codes for the protein cystatin F, which inhibits phagocytosis. However, stimulation of TREM2 with phosphatidylserine‐exposing targets causes increased phagocytosis of these targets specifically, with R47H TREM2 being stimulated more than WT TREM2. This results in increased phagocytic uptake of phosphatidylserine‐exposing targets by microglia expressing R47H TREM2 compared to microglia expressing WT TREM2, leading to increased loss of neurons and synapses by R47H TREM2 expressing microglia.

## Data Availability

The data used in this study are available from the corresponding authors upon request.
