# Supplementary figures and images for: Alzheimer's disease‐associated R47H TREM2 increases, but wild‐type TREM2 decreases, microglial phagocytosis of synaptosomes and neuronal loss
Source: Glia. 2022 Dec 8;71(4):974–90. doi: 10.1002/glia.24318 (PMC10952257; doi:10.1002/glia.24318)

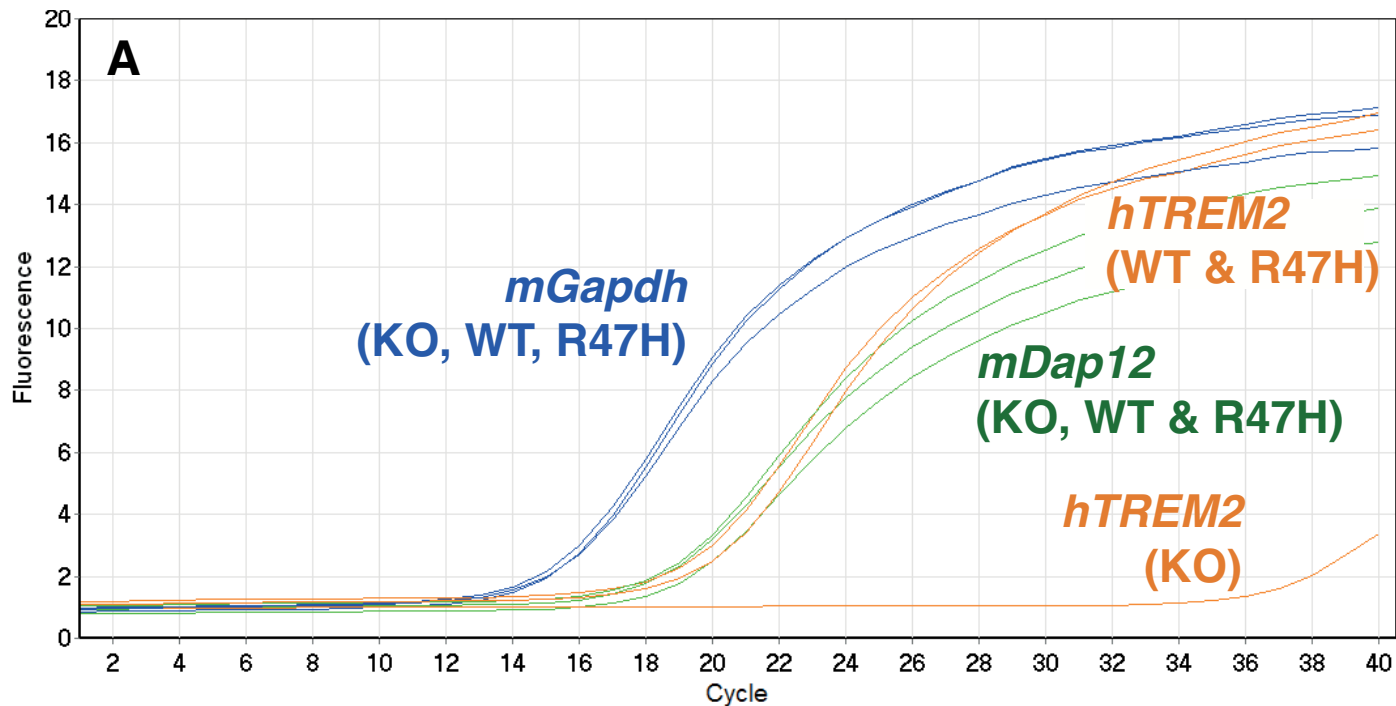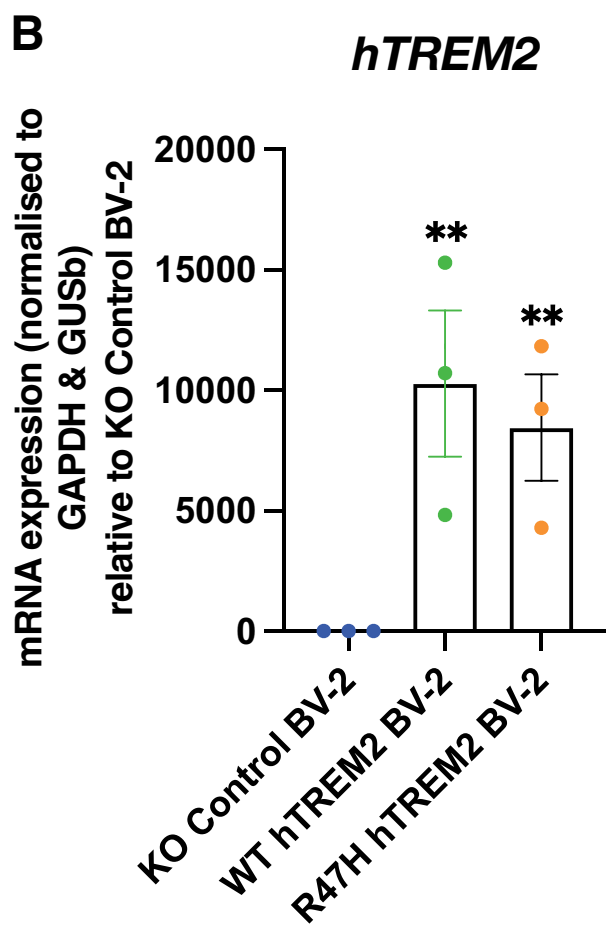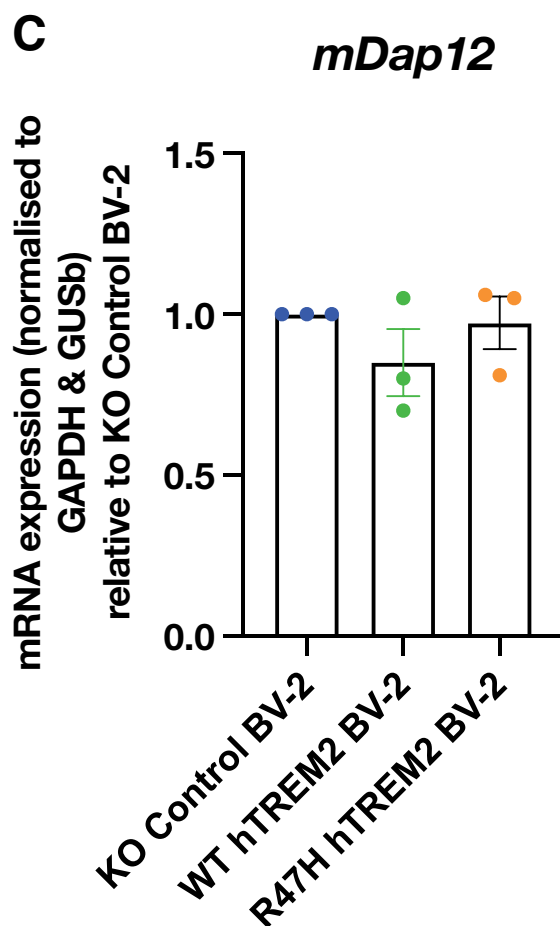

Supplement: Supplementary file 1 — FIGURE S1. TREM2 & DAP12 expression in BV‐2 cells. Stable BV‐2 microglia cell‐lines were created using lentiviral transduction with plasmids containing gene of interest and eGFP. (a, b, c) RT‐qPCR using RNA from mTREM2 KO BV‐2 microglia transduced with either control vector (KO), hTREM2 WT (WT) or hTREM2 R47H (R47H). (a) RT‐qPCR amplification curves showing expression of mGapdh (blue), mDap12 (green) and hTREM2 (orange). Take‐off points for hTREM2 and mDap12 are comparable for hTREM2 WT and hTREM2 R47H BV‐2 cells. (b, c) Expression is normalized to the geometric mean of housekeepers Gapdh & Gusb and fold changes are shown relative to KO Control BV‐2. Error bars represent SEM, ordinary one‐way ANNOVA performed on raw comparative concentration values not normalized to KO Control BV‐2 data. Brown‐Forsythe and Welch post hoc test applied. KO Control BV‐2 versus WT hTREM2 BV‐2 **P = .0027, KO Control BV‐2 versus R47H hTREM2 BV‐2 **P = .0076 [file GLIA-71-974-s012.pdf]

**A****TREM2 Surface Expression**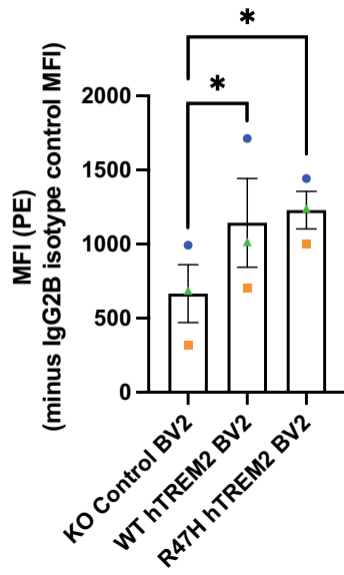**B****KO Control BV-2**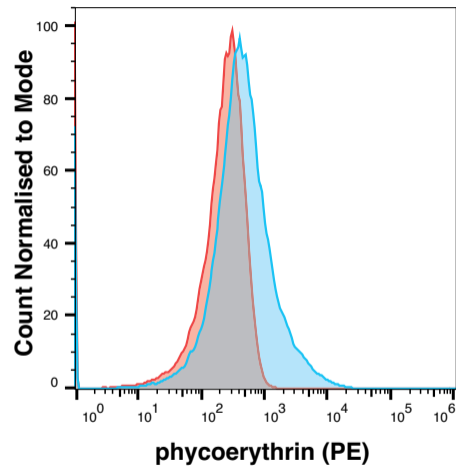**WT hTREM2 BV-2**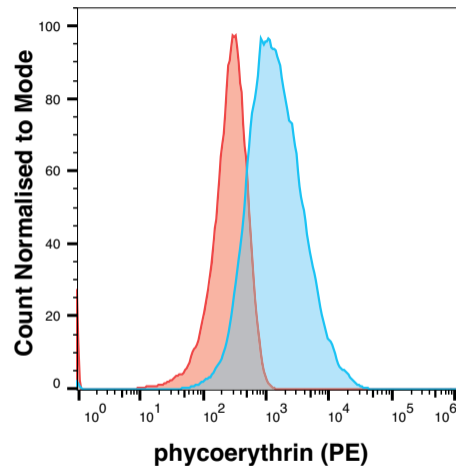**R47H hTREM2 BV-2**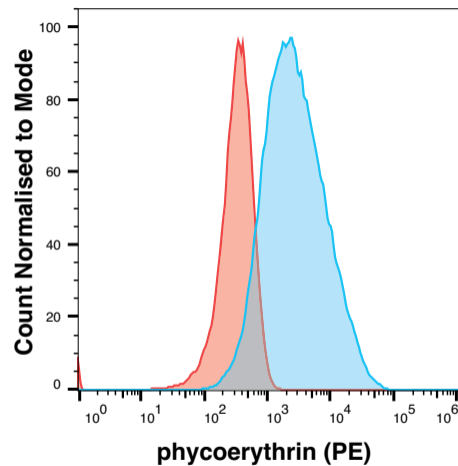

Supplement: Supplementary file 2 — FIGURE S2. Expression of TREM2 protein on surface of BV‐2 microglia cell‐lines. (a) MFI (PE) values for KO Control BV‐2, WT hTREM2 BV‐2 & R47H hTREM2 BV‐2 using rat anti‐human/mouse TREM2 antibody (clone: 237920) with corresponding MFI values for rat IgG2B isotype control antibody (clone: 141945) subtracted (N = 3). Error bars represent S.E.M, paired one‐way ANNOVA performed, followed by Tukey's post hoc test. KO Control BV‐2 versus WT hTREM2 BV‐2 *P = .0468, KO Control BV‐2 versus R47H hTREM2 BV‐2 *P = .0276. (b) Representative histograms of TREM2 protein expression on the surface of the three BV‐2 cell lines; KO Control BV‐2, WT hTREM2 BV‐2 & R47H hTREM2 BV‐2. Histogram for isotype control antibody shown in red and rat anti‐human/mouse TREM2 shown in blue. Both antibodies are conjugated to phycoerythrin (PE) and so a shift in the PE channel represents increased TREM2 expression on surface of microglia. [file GLIA-71-974-s007.pdf]

# BV-2 Proliferation

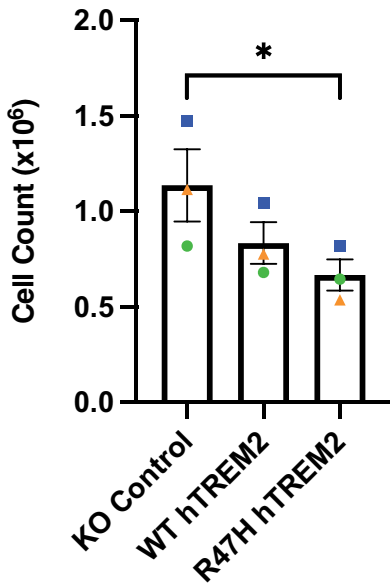

Supplement: Supplementary file 3 — FIGURE S3. TREM2 expression mildly reduces proliferation. Overexpression of TREM2 appears to slightly decrease BV‐2 microglia proliferation after 24 h, with R47H TREM2 significantly decreasing proliferation versus KO Control BV‐2 cells. 500,000 BV‐2 cells were seeded at in DMEM supplemented with 10% heat‐inactivated FBS and 1% Pen/Strep for 24 h after which proliferation was measured by cell density (counted using hemocytometer). N = 3. Data points with the same color/shape represent paired data carried out in the same experimental repeat. Error bars represent SEM, paired one‐way ANNOVA performed, followed by Tukey's post hoc test. KO Control BV‐2 versus R47H hTREM2 BV‐2 *P = .0249 [file GLIA-71-974-s006.pdf]

**Synaptosomes only (blue) vs Synaptosomes + PE/Dazzle™-conjugated annexin-V (red)**

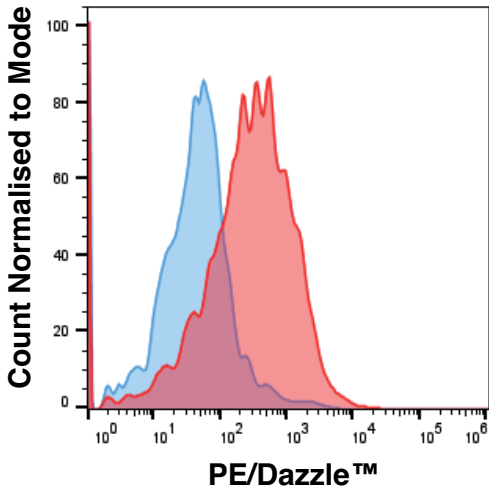

Supplement: Supplementary file 4 — FIGURE S4. Phosphatidylserine exposure on synaptosomes shown using PE/Dazzle™ conjugated Annexin‐V. Synaptosomes were combined with PE/Dazzle™‐annexin V which binds to exposed phosphatidylserine. Synaptosomes were then analyzed via flow‐cytometry. Shift of red histogram (synaptosomes labeled with PE/Dazzle™ annexin V) versus blue histogram (synaptosomes without labeling) in the PE/Dazzle™ channel represents population of synaptosomes exposing phosphatidylserine. [file GLIA-71-974-s004.pdf]

**IB4**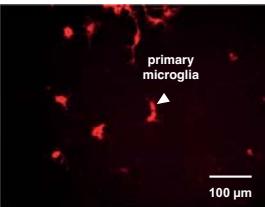**Hoechst**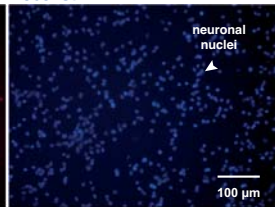**NeuO**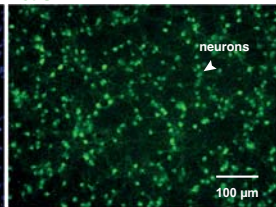**MERGE: IB4, Hoechst, NeuO**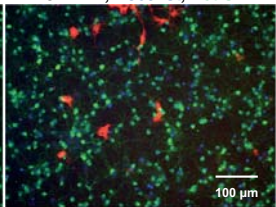**PHASE**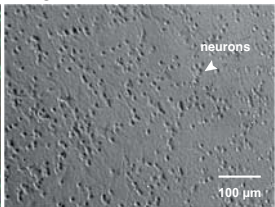

Supplement: Supplementary file 6 — FIGURE S6. Primary cerebellar mouse neuronal‐glial cultures stained with live neuronal marker. Cultures were stained with a marker of live‐neurons NeuO (green), a microglial marker IB4 (red) and nuclear marker Hoechst (blue). These images are shown alongside a composite image of the three fluorescence channels and alongside the corresponding phase image. NeuO was used to validate the used method of distinguishing nuclei using nuclear morphology and an absence of IB4 staining. [file GLIA-71-974-s010.pdf]

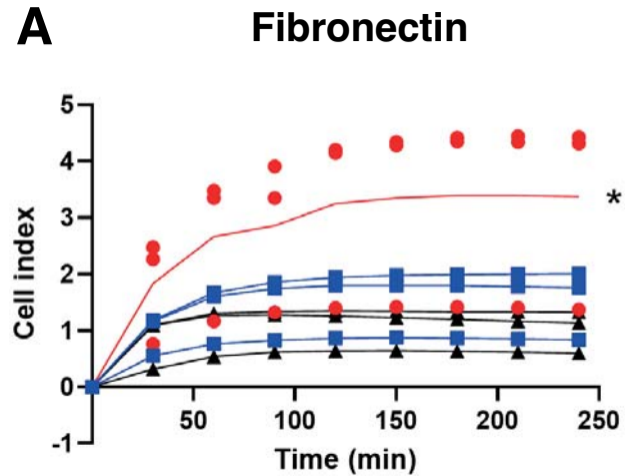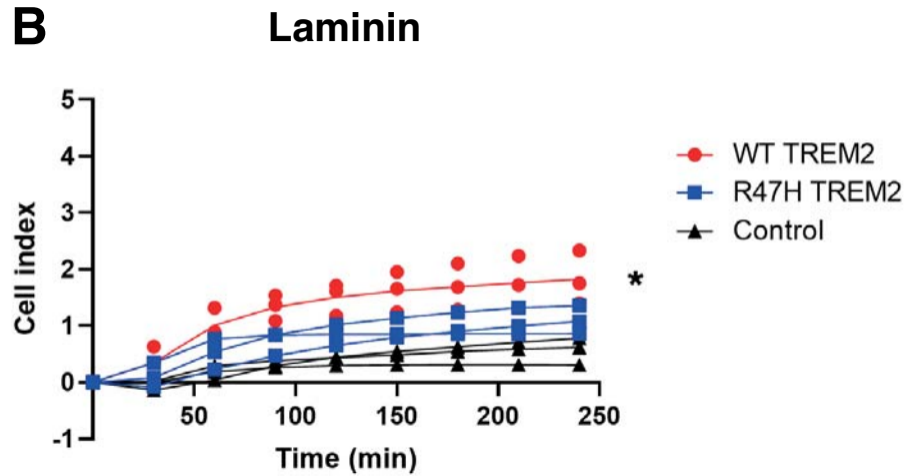

Supplement: Supplementary file 8 — FIGURE S8. Overexpression of TREM2 (WT and R47H mutant) modulates attachment to laminin and fibronectin and proliferation. (a) WT TREM2 increases attachment to 20 μg/ml laminin over 4 h (b) WT TREM2 increases attachment to 10 μg/ml fibronectin over 240 min. Attachment was measured by electrical impedance of cell layer. N = 3. Statistics: repeated measures one way ANOVA, followed by Tukey's post hoc test, on end point (240 min) value. * is compared to control. *p < .05. [file GLIA-71-974-s001.pdf]

# Proliferation of cells

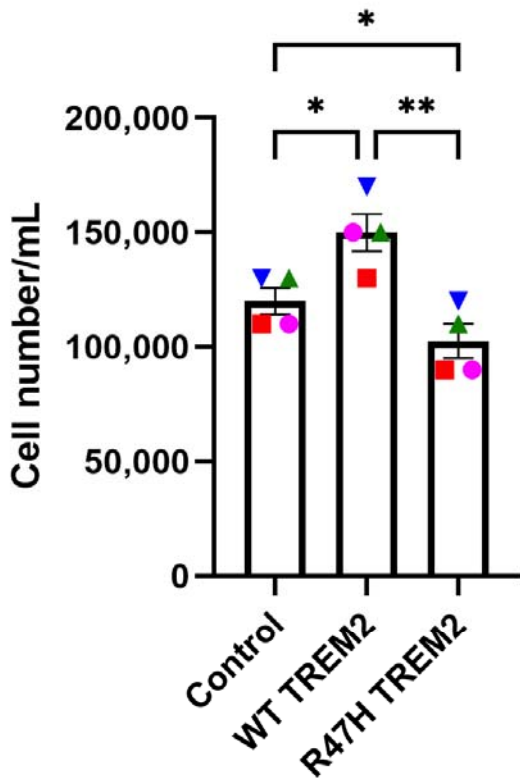

Supplement: Supplementary file 9 — FIGURE S9. TREM2 expression mildly modulates proliferation in CHME‐3 cells. WT TREM2 increases, while R47H mutant decreases, proliferation of CHME‐3 cells after seeding the same density of cells in presence of DMEM supplemented with 10% heat‐inactivated FBS and 1% antibiotics for 48 h. Proliferation measured by cell density (counted using hemocytometer). N = 4. Repeats done on the same day with the three variant cell lines are depicted by the same color and symbols. Statistics: repeated one‐way ANOVA, followed by Tukey's post hoc test. *p < .05, **p < .01. [file GLIA-71-974-s005.pdf]

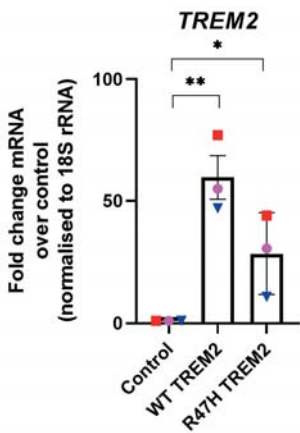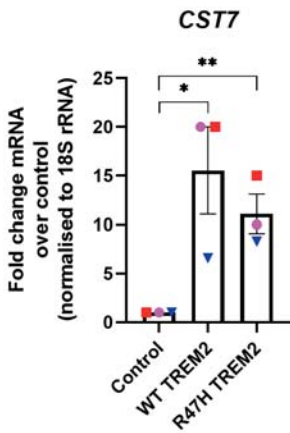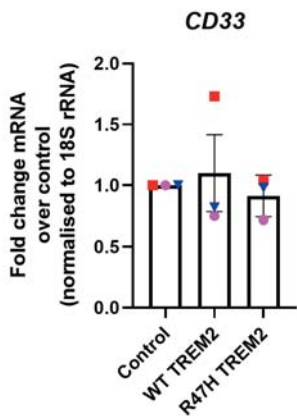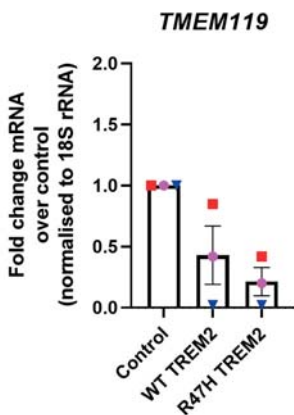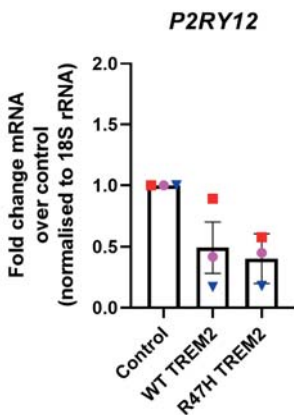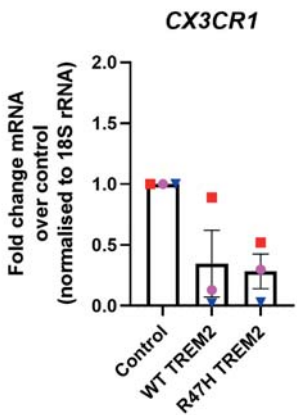

Supplement: Supplementary file 10 — FIGURE S10. CST7 mRNA expression is significantly increased in WT and R47H TREM2 overexpressing CHME‐3 cells. qPCR analysis of key microglial genes indicative of disease associated microglia (DAM). CST7 is significantly upregulated in TREM2 overexpressing CHME‐3 cells. CD33, TMEM119, P2RY12 and CX3CR1 mRNA levels are not significantly changed between the different cell lines. However, there is a trend toward reduced levels of homeostatic markers TMEM119, P2RY12 and CX3CR1. Data are expressed as fold change over the control (EGFP only) cell line and all samples are normalized to 18 S rRNA housekeeping gene. N = 3. Repeats done on the same day with the three variant cell lines are depicted by the same color and symbols. Statistics: ratio t‐test comparing control to WT TREM2 or R47H TREM2 separately. *p < .05, **p < .01. [file GLIA-71-974-s002.pdf]

**A**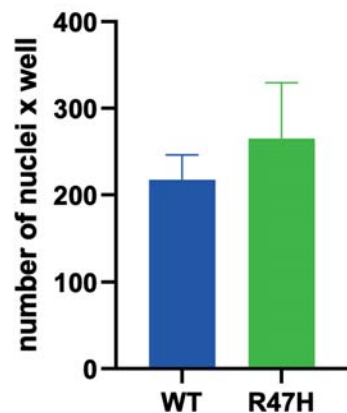**B**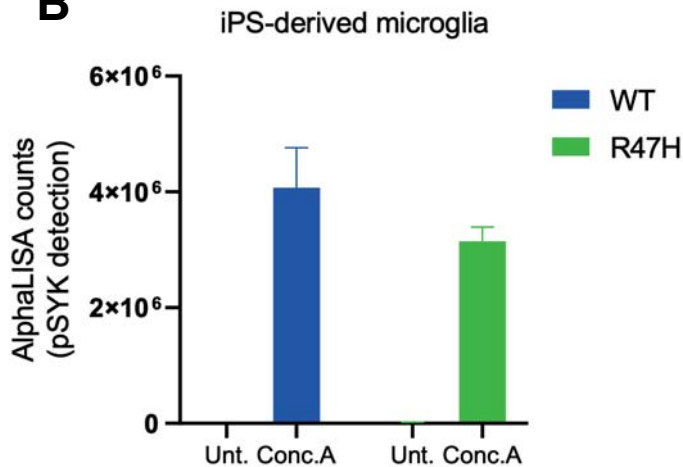**C**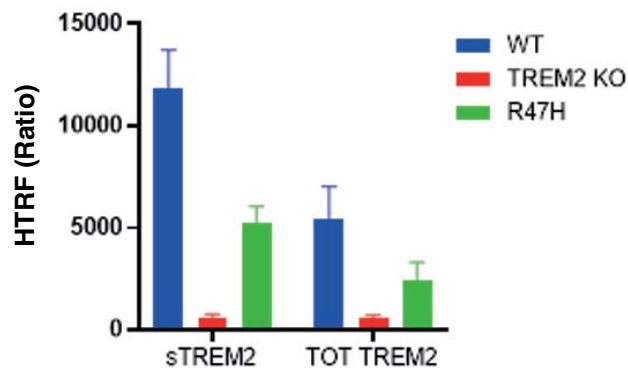

Supplement: Supplementary file 11 — FIGURE S11. Comparison of (a) number of cells, (b) pSYK activation by concanavalin A, and (c) amount of sTREM2 and total TREM2, in wild‐type and R47H TREM2 expressing hiPSC‐derived microglia. (a) iPSC‐Mg were seeded and maturated in 384‐well plate. Homogeneity of cell seeding was confirmed by counting nuclei for both WT and R47H at the end of maturation. Data shown represent mean ± SD. (b) Comparable TREM2‐independent activation of SYK is detectable in both WT and TREM2‐R47H IPSC‐Mg after treatment with 500 ng/ml Concanavalin A. Data shown represent mean ± SD. (c) Soluble TREM2 (sTREM2) and cellular TREM2 (TOT TREM2) were quantified by Homogeneous Time Resolved Fluorescence (HTRF) using anti‐TREM2 antibodies to probe the cellular supernatant or cell lysates. [file GLIA-71-974-s009.pdf]

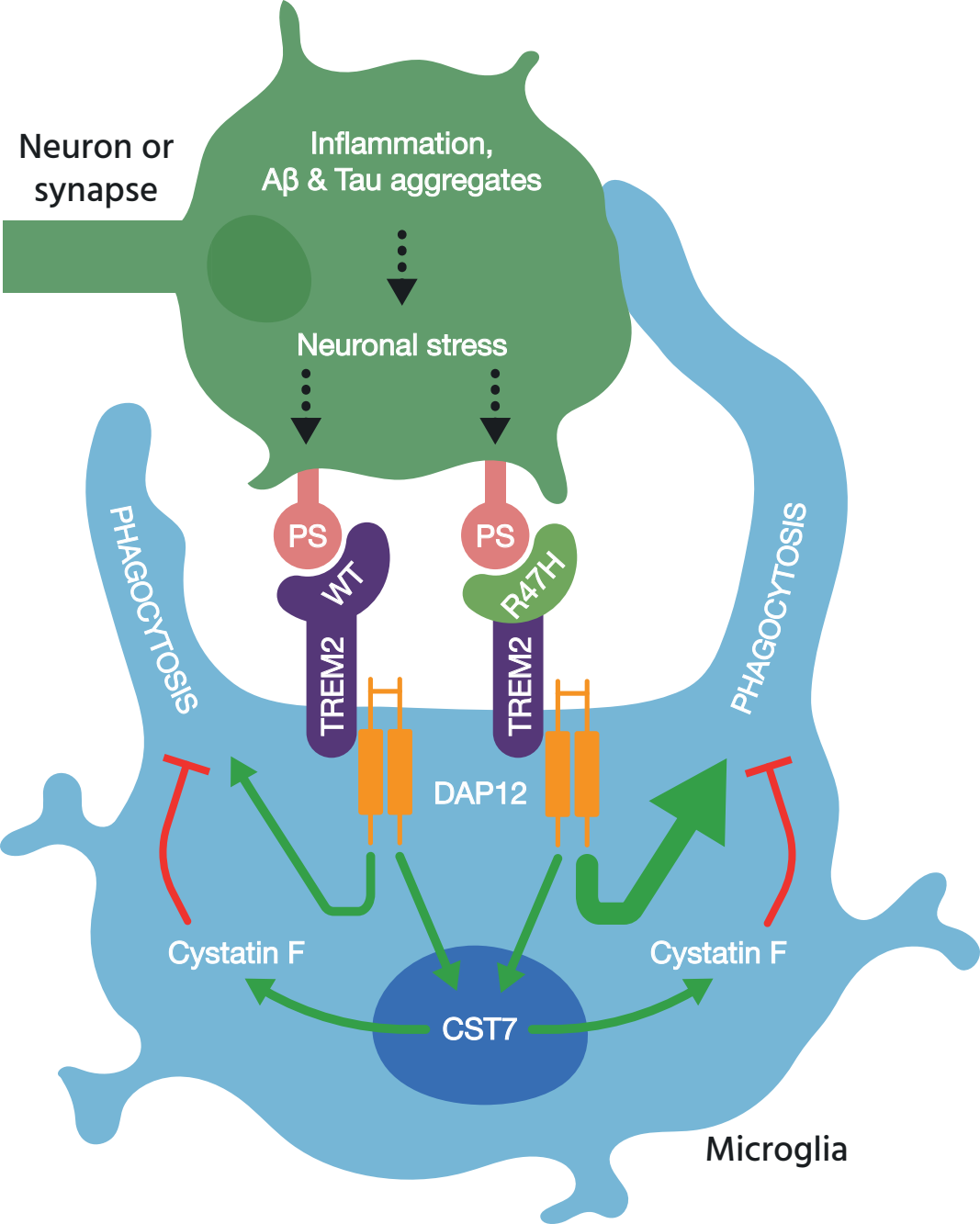

Supplement: Supplementary file 12 — FIGURE S12. Summary of findings and potential means by which R47H TREM2 potentiates Alzheimer's disease. Activation of TREM2 on microglia may have two opposing effects on the phagocytosis of synaptosomes and other phosphatidylserine‐exposing targets: (i) a stimulation due to TREM2 acting as a phagocytic receptor for these targets, and (ii) an inhibition due to the induced expression of CST7. Which of these opposing effects predominates is likely to depend on conditions such as time course, phosphatidylserine exposure and TREM2 variant. Stimulation of both WT and R47H TREM2 on microglia induces expression of CST7, a disease associated microglia (DAM) gene that codes for the protein cystatin F, which inhibits phagocytosis. However, stimulation of TREM2 with phosphatidylserine‐exposing targets causes increased phagocytosis of these targets specifically, with R47H TREM2 being stimulated more than WT TREM2. This results in increased phagocytic uptake of phosphatidylserine‐exposing targets by microglia expressing R47H TREM2 compared to microglia expressing WT TREM2, leading to increased loss of neurons and synapses by R47H TREM2 expressing microglia. [file GLIA-71-974-s008.pdf]
