# Supplementary material for: Alzheimer's disease‐associated R47H TREM2 increases, but wild‐type TREM2 decreases, microglial phagocytosis of synaptosomes and neuronal loss
Source: Glia. 2022 Dec 8;71(4):974–90. doi: 10.1002/glia.24318 (PMC10952257; doi:10.1002/glia.24318)
Supplement: Supplementary file 5 — FIGURE S5. Representative phase and merged images of primary cerebellar mouse neuronal‐glial cultures co‐cultured with KO Control, WT hTREM2 BV‐2 or R47H hTREM2 BV‐2 microglia ± annexin‐V for 24 h. Phase images for each condition are shown alongside their corresponding composite image of the phase channel and three fluorescent channels; red (PI‐positive necrotic cells), green (IB4‐positive microglia and eGFP expressing BV‐2 microglia) and blue (nuclei stained with Hoechst). Neurons were distinguished from astrocytes and microglia through their nuclear morphology and absence of IB4 staining. [file GLIA-71-974-s003.pdf]

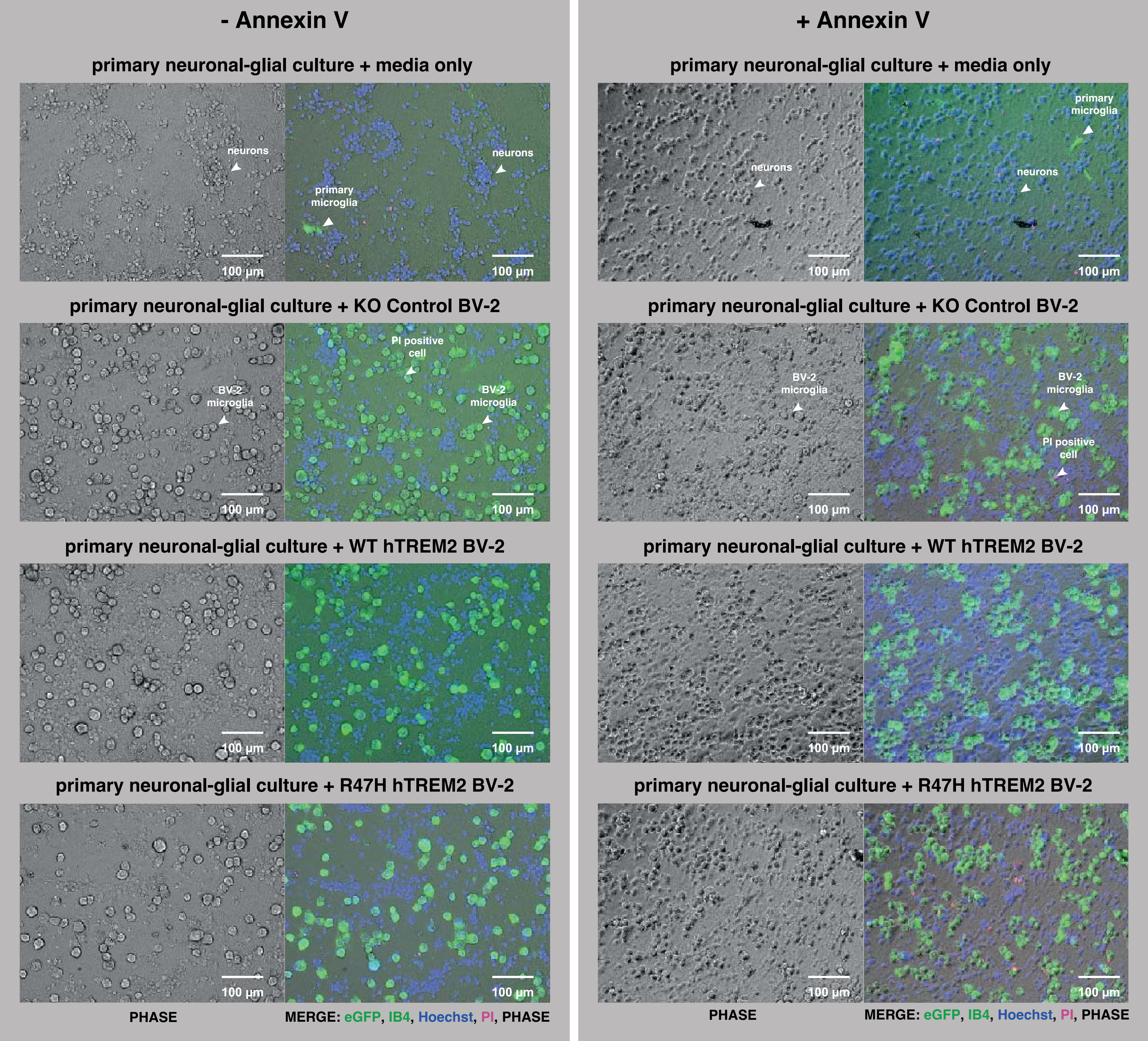

- Annexin V
+ Annexin V
primary neuronal-glial culture + media only
neurons
primary microglia
100 µm
primary neuronal-glial culture + KO Control BV-2
BV-2 microglia
PI positive cell
primary neuronal-glial culture + WT hTREM2 BV-2
primary neuronal-glial culture + R47H hTREM2 BV-2
PHASE
MERGE: eGFP, IB4, Hoechst, PI, PHASE
