# Supplementary material for: Alzheimer's disease‐associated R47H TREM2 increases, but wild‐type TREM2 decreases, microglial phagocytosis of synaptosomes and neuronal loss
Source: Glia. 2022 Dec 8;71(4):974–90. doi: 10.1002/glia.24318 (PMC10952257; doi:10.1002/glia.24318)
Supplement: Supplementary file 7 — FIGURE S7. TREM2 & DAP12 expression in CHME‐3 cells at mRNA level and expression of TREM2 protein on CHME‐3 cell surface. qPCR analysis of CHME‐3 cells expressing (a) human TREM2 and (b) human DAP12. (c) Human TREM2 protein expression on cell surface of CHME‐3 cells. (d–f) flow plots of CHME‐3 cells expressing TREM2 in (d) in control cell line (EGFP only), (e) WT TREM2 overexpressing cell line, and (f) R47H TREM2 overexpressing cell line. Data shown are for one biological repeat. Red histogram = monoclonal rat IgG2B isotype control antibody (clone: 141945), blue histogram = rat anti‐human/mouse TREM2 (clone: 237920). All antibodies were conjugated to phycoerythrin (PE). [file GLIA-71-974-s011.pdf]

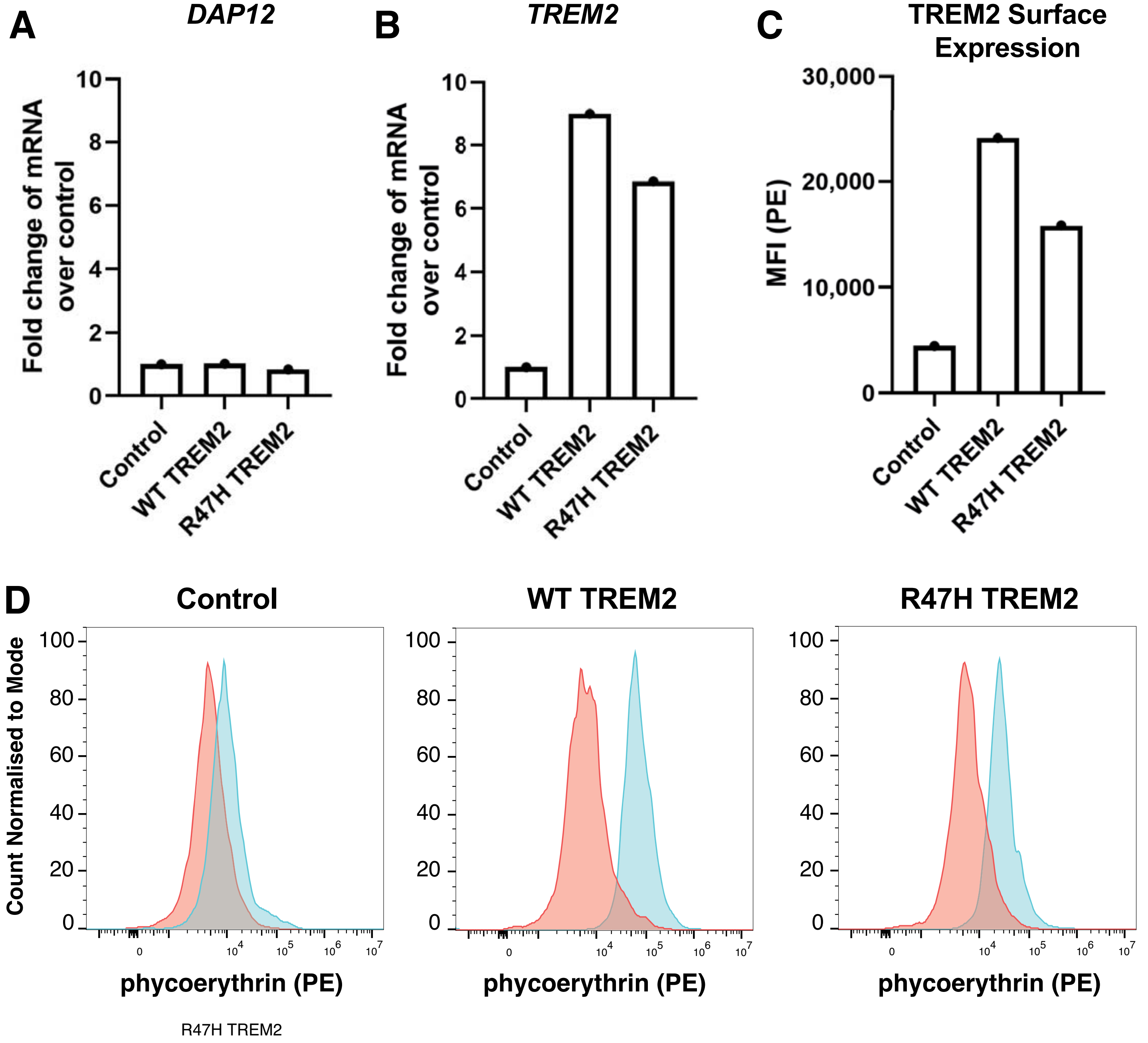
A
DAP12
Fold change of mRNA over control
10
8
6
4
2
0
Control
WT TREM2
R47H TREM2
B
TREM2
Fold change of mRNA over control
10
8
6
4
2
0
Control
WT TREM2
R47H TREM2
C
TREM2 Surface Expression
MFI (PE)
30,000
20,000
10,000
0
Control
WT TREM2
R47H TREM2
D
Control
WT TREM2
R47H TREM2
Count Normalised to Mode
100
80
60
40
20
0
phycoerythrin (PE)
R47H TREM2
